# Discovery of a natural small-molecule AMP-activated kinase activator that alleviates nonalcoholic steatohepatitis

**DOI:** 10.1007/s42995-023-00168-z

**Published:** 2023-04-30

**Authors:** Jin Chen, Li Xu, Xue-Qing Zhang, Xue Liu, Zi-Xuan Zhang, Qiu-Mei Zhu, Jian-Yu Liu, Muhammad Omer Iqbal, Ning Ding, Chang-Lun Shao, Mei-Yan Wei, Yu-Chao Gu

**Affiliations:** 1 Key Laboratory of Marine Drugs, the Ministry of Education, School of Medicine and Pharmacy, Ocean University of China, Qingdao, 266003 China; 2 Laboratory for Marine Drugs and Bioproducts, Laoshan Laboratory, Qingdao, 266237 China; 3 College of Food Science and Engineering, Ocean University of China, Qingdao, 266003 China; 4 Key Laboratory of Glycoscience and Glycotechnology of Shandong Province, Qingdao, 266003 China; 5 The Massachusetts General Hospital Cancer Center, Harvard Medical School, Boston, MA 02114 USA

**Keywords:** NASH, AMPK, Inflammation, Lipid metabolism, Fibrosis, Marine drug

## Abstract

Non-alcoholic steatohepatitis (NASH) is a primary cause of cirrhosis and hepatocellular carcinoma. Unfortunately, there is no approved drug treatment for NASH. AMP-activated kinase (AMPK) is an important metabolic sensor and whole-body regulator. It has been proposed that AMPK activators could be used for treating metabolic diseases such as obesity, type 2 diabetes and NASH. In this study, we screened a marine natural compound library by monitoring AMPK activity and found a potent AMPK activator, candidusin A (**CHNQD-0803**). Further studies showed that **CHNQD-0803** directly binds recombinant AMPK with a *K*_D_ value of 4.728 × 10^–8^ M and activates AMPK at both molecular and intracellular levels. We then investigated the roles and mechanisms of **CHNQD-0803** in PA-induced fat deposition, LPS-stimulated inflammation, TGF-β-induced fibrosis cell models and the MCD-induced mouse model of NASH. The results showed that **CHNQD-0803** inhibited the expression of adipogenesis genes and reduced fat deposition, negatively regulated the NF-κB-TNFα inflammatory axis to suppress inflammation, and ameliorated liver injury and fibrosis. These data indicate that **CHNQD-0803** as an AMPK activator is a novel potential therapeutic candidate for NASH treatment.

**Supplementary Information:**

The online version contains supplementary material available at 10.1007/s42995-023-00168-z.

## Introduction

Non-alcoholic fatty liver disease (NAFLD) is the main cause of cirrhosis and hepatocellular carcinoma worldwide, affecting 25% of global adults (Powell et al. 2021). NASH is an inflammatory form of NAFLD, which is characterized by fibrosis progression and hepatocyte damage that ultimately leads to cirrhosis (Chalasani et al. 2018). Although extensive basic and clinical research has been carried out, therapeutic options for NASH are limited (Cai et al. 2019). Studies have shown that the most important feature of the pathogenesis of NASH is driven by fibrosis, which is a complex process caused by chronic inflammatory states associated with oxidative stress, hepatic steatosis, insulin resistance, and obesity (Apostolopoulou et al. 2018; Bril et al. 2017; Gancheva et al. 2018; Drenth and Schattenberg 2020; Samuel and Shulman 2018). Targeted intervention for pathogenesis is the current hotspot of drug research and development.

The potential NASH drug targets include peroxisome proliferator-activated receptor (PPAR), TGF-β1/Smad2, nuclear factor-κB (NF-κB), farnesoid X receptor (FXR), THR-β and MAPK Signal pathways (Kannt et al. 2021; Li et al. 2021; Liu et al. 2021). A variety of candidate drugs are being tested against these targets, the most advanced one is obeticholic acid (OCA) (Sun et al. 2021). Despite demonstrating a significant improvement in liver fibrosis and reaching the primary endpoint, the clinical trial of OCA failed due to safety issues (pruritus, dyslipidemia) (Taylor et al. 2020). In addition, most of the candidate drugs developed for other targets have also ended in failure (Drenth and Schattenberg 2020). Therefore, it is generally accepted that NASH requires multi-target combinatory therapy (Rotman and Sanyal 2017), and it might be an effective strategy to target the key nodes of signaling pathways associated with NASH.

AMPK, as a metabolic stress sensor, plays a critical role in adaptive response to falling energy levels resulting from heat shock, ischemia, hypoxia, and glucose deprivation levels (Hardie and Carling 1997; Steinberg and Kemp 2009). Some studies reported that AMPK modulates adipose tissue fatty acid synthesis and oxidation and attenuates hepatic steatosis (Scheja and Heeren 2016; Mottillo et al. 2016). Also, AMPK attenuates inflammation in different conditions (O'Neill and Hardie 2013; Huang et al. 2015) and reduces hepatic fibrosis via multiple mechanisms, including proliferation, preventing HSC activation, inhibiting fibrogenic gene expression and migration (Jian et al. 2020; Zhao et al. 2020), and ultimately alleviating the development of NASH. AMPK inhibits NASH through multiple pathways, therefore, AMPK appears to be a potential candidate for multi-target therapy of NASH.

Due to the potential effects of AMPK activities on NASH, research on AMPK activators has received extensive attention. The first allosteric AMPK activator, A-769662, activates AMPK by modulating the β subunit (Cool et al. 2006; Hawley et al. 2012). However, its poor bioavailability prevented clinical trials. PT1, another allosteric AMPK activator, inhibits autoinhibition in the α subunit and exerts some cellular effects. Unfortunately, PT1 failed to improve metabolic function in animal models, due to poor bioavailability and bioactivity (Pang et al. 2008). Therefore, developing effective AMPK activators is an urgent unmet need for the treatment of NASH.

Natural products are an essential source of new chemical and biological diversity. The oceans are a valuable source of products with significant antimicrobial, anti-inflammatory, antiviral, antitumor, antimalarial, and anti-oxidant properties, thanks to their unique aquatic environments and rich biodiversity (Hou et al. 2019). A variety of clinical drugs and drug candidates from the ocean have already been used or are under investigation, including the first anticancer drug trabectedin and the marine peptide ziconotide for pain (Molinski et al. 2009).

Based on the screening of the marine natural compound library in our laboratory, we identified one *p*-terphenyl candidusin A (**CHNQD-0803**) and showed that this compound directly binds and activates AMPK. Our studies showed that **CHNQD-0803** can effectively decrease lipid synthesis, alleviate cellular inflammation, and improve hepatocyte fibrosis. Thus, **CHNQD-0803** is a well-characterized allosteric AMPK activator and a valuable small molecule with promising efficacy for more specific treatment of liver fibrosis and hepatic steatosis inflammation.

## Results

### Discovery of CHNQD-0803 as an activator of AMPK

**Fig. 1:**
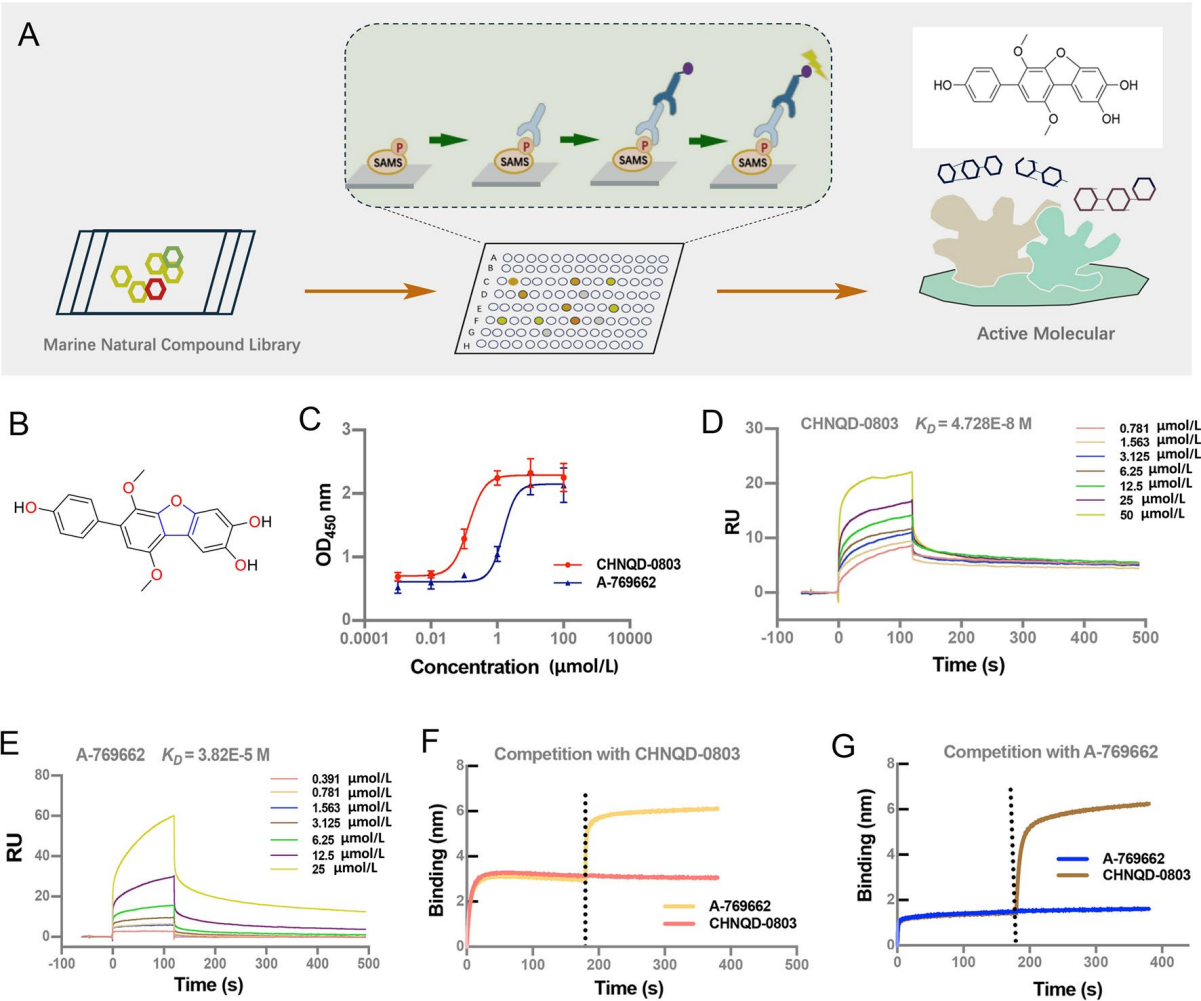
Structure, activity and affinity of CHNQD-0803 for AMPK. **A** A schematic diagram of screening of AMPK activation. **B** The chemical structure of **CHNQD-0803**. **C CHNQD-0803** allosterically activates recombinant α1β1γ1 with EC_50_ value of 0.14 μmol/L. A-769662 allosterically activates recombinant α1β1γ1 with EC_50_ value of 1.53 μmol/L. **D** Dissociation equilibrium constants (*K*_*D*_) of **CHNQD-0803** were determined using Biacore. **E** Dissociation equilibrium constants (*K*_*D*_) of A-769662 were determined using Biacore. Results from at least three independent experiments, data are expressed as mean ± SD. **F**, **G** To test the competitive ability of different molecules to bind AMPK, competition assays were performed by using BLI assays. Immobilized AMPK was first saturated with **CHNQD-0803** (**F**) or A-769662 (**G**). Then adding the second compound in the presence of the first molecule, further shifts were measured to determine how well the second molecule bound to the antigen. The black dotted line indicates the second molecule binding to the antigen. The continued increase in binding capacity after the addition of the second molecule indicates that there is no competitive binding between the two molecules

We screened the marine natural compound library of our lab by monitoring the phosphorylation level of SAMS (AMP-activated protein kinase substrate) peptide with ELISA (Fig. 1A) and identified **CHNQD-0803** as a potential activator of AMPK (Fig. 1B). Next, we tested the half-maximal effective concentration (EC_50_) value of **CHNQD-0803** on recombinant AMPK and the results showed that the EC_50_ value is 0.14 μmol/L (Fig. 1C). Moreover, the surface plasmon resonance (SPR) assays indicated that the dissociation equilibrium constant (*K*_D_ value) of AMPK and **CHNQD-0803** complex is 4.728 × 10^–8^ mol/L (Fig. 1D). A-769662 is a well-studied AMPK allosteric activator, which activates AMPK by affecting the carbohydrate-binding module at the N-terminus of β subunit and residues from γ subunit (Xiao et al. 2013). We showed that the EC_50_ value and *K*_D_ value of A-769662 is 1.53 μmol/L and 3.82 × 10^–5^ mol/L, respectively (Fig. 1C and E).

In order to explore the binding site of **CHNQD-0803** on AMPK, we first performed a compete binding experiment of **CHNQD-0803** and A-769662 for binding to AMPK by biolayer interferometry assays (BLI). An AMPK analyte in solution was saturated on the biosensors labeled with NTA, and then a second molecule was added while the first was present. As shown, **CHNQD-0803** did not compete with A-769662 for binding to AMPK (Fig. 1F and G). The result suggests that **CHNQD-0803** binds epitopes on AMPK different than A-769662; thus, there may be a different mechanism in activating AMPK. In summary, these results show that **CHNQD-0803** binds AMPK with higher affinity and may be a better choice for the activator of AMPK.

### Structure−activity relationships of CHNQD-0803 as an AMPK activator

**Fig. 2:**
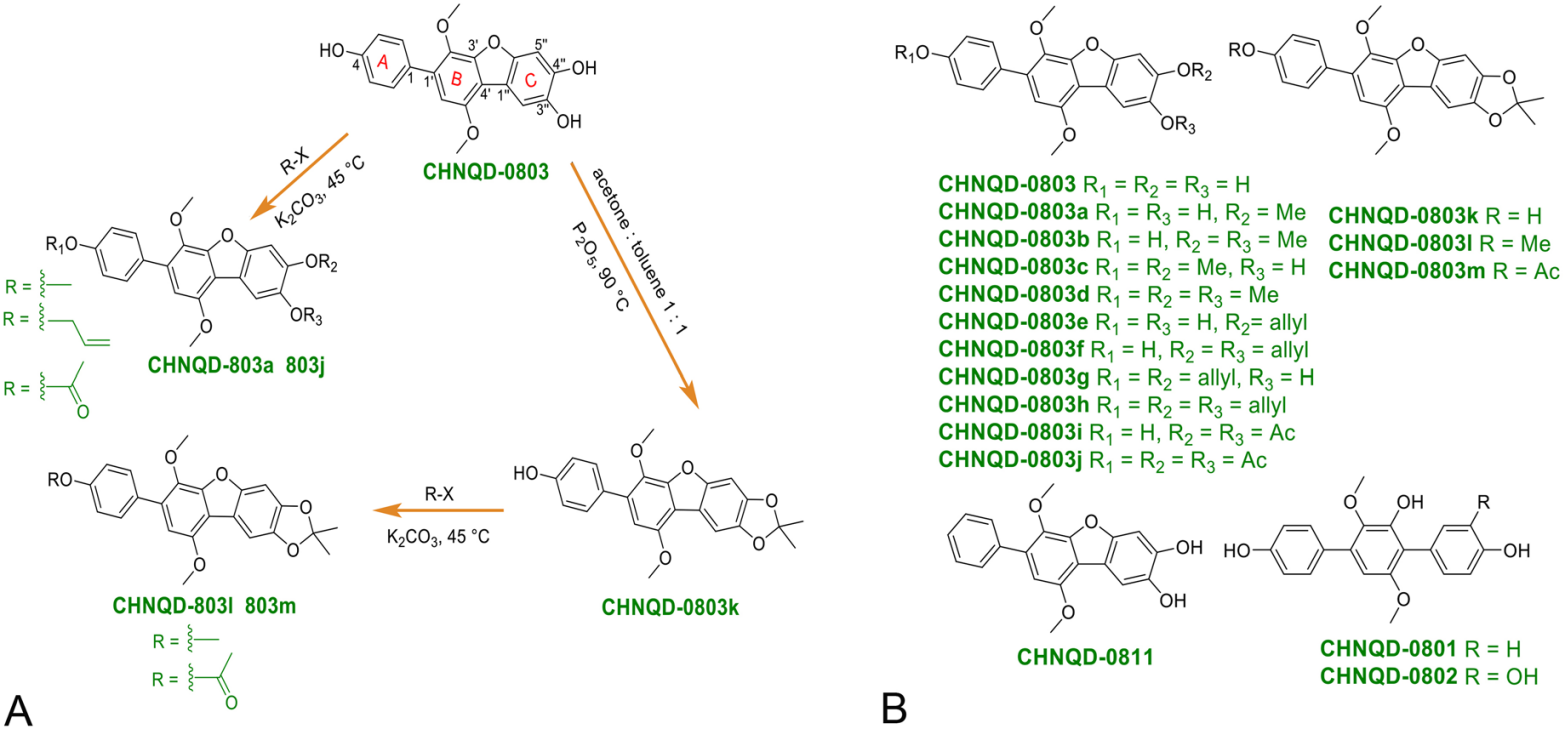
Structures and general semisynthetic strategy of compounds

**Fig. 3:**
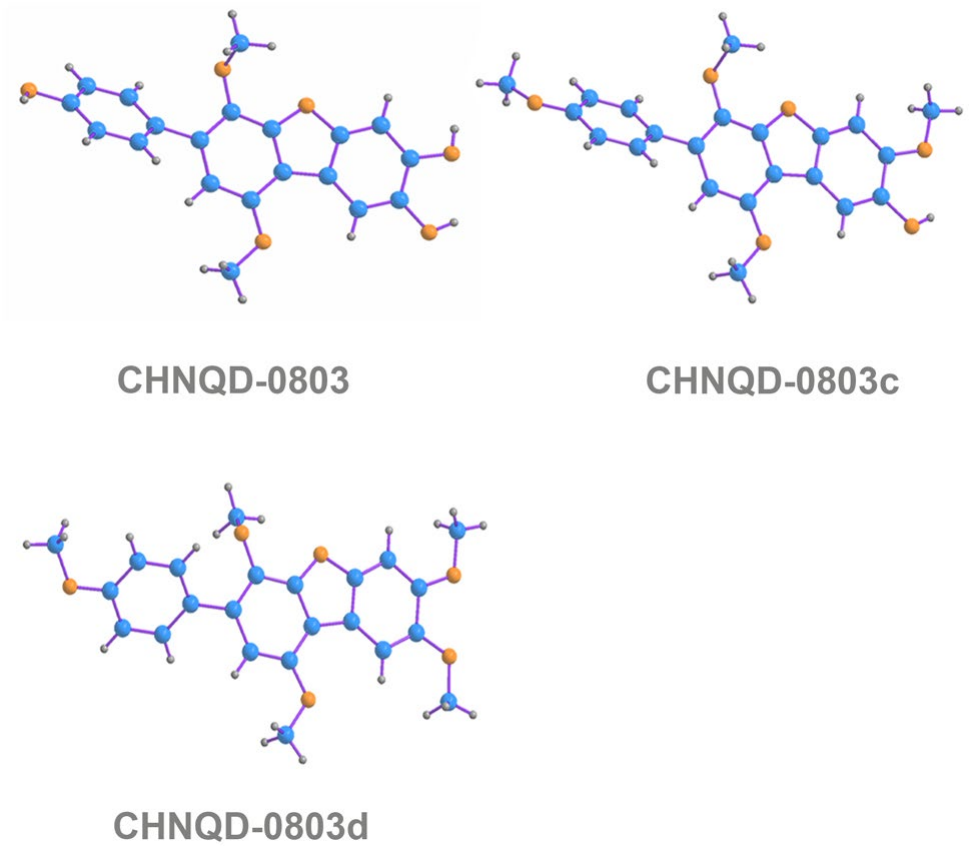
X-ray crystallographic structure of CHNQD-0803, CHNQD-0803c and CHNQD-0803d

**Fig. 4:**
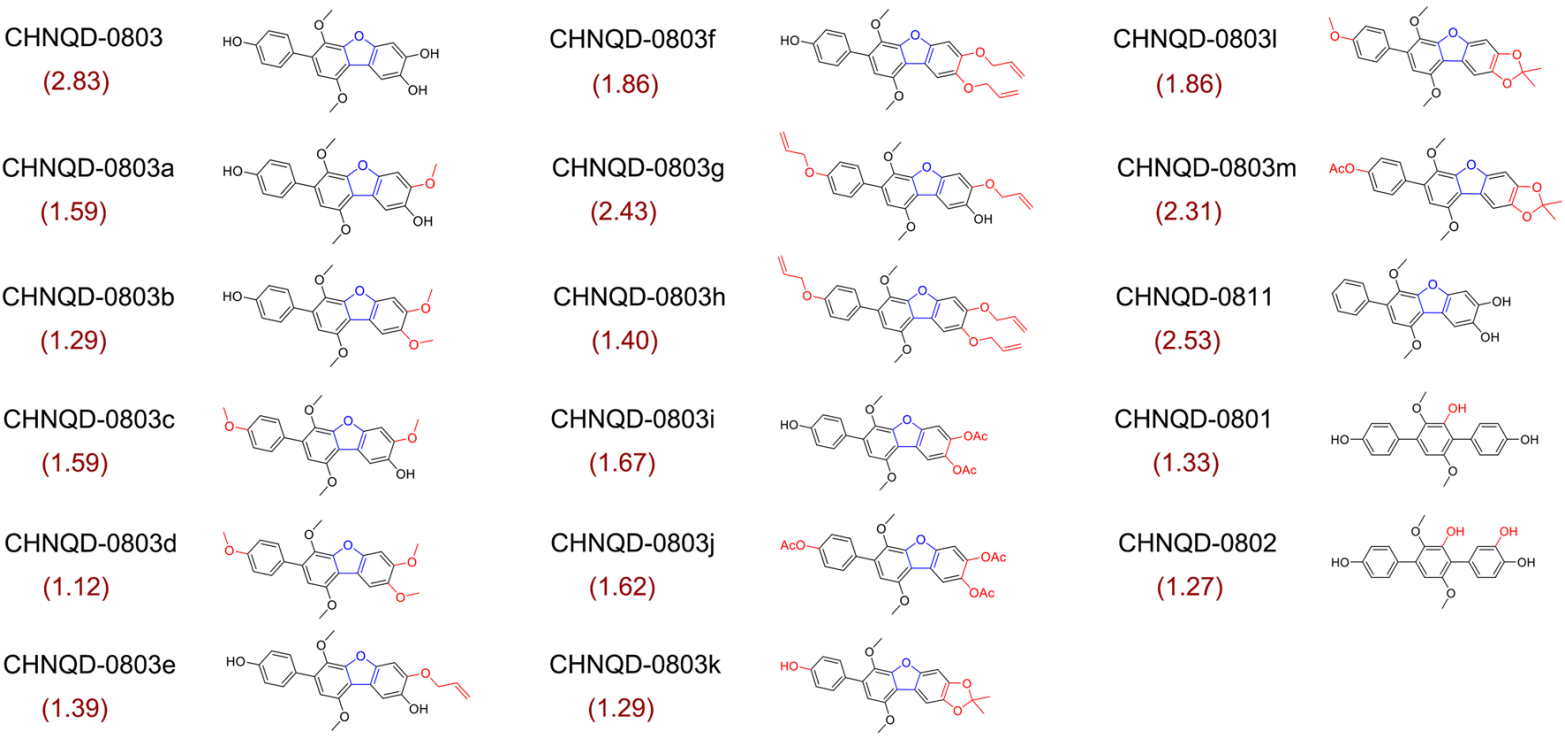
AMPK kinase activity treated with CHNQD-0803 and its derivatives in HepG2 cells. The values in parentheses are the activation fold of the compound

To further explore the structure–activity relationship of **CHNQD-0803** as an AMPK activator, a series of derivatives of **CHNQD-0803** were semi-synthesized to increase the chemical diversity for biological evaluation. A group of substituents at 4, 3′′′or 4′′′-hydroxyl groups of aromatic ethers **CHNQD-0803a–h** were semi-synthesized through the Williamson ether synthesis. Acylation reaction was carried out by reaction of **CHNQD-0803** with acetyl chloride to afford **CHNQD-0803i** and **CHNQD-0803h**. The derivative **CHNQD-0803k** was prepared with P_2_O_5_ in a solvent of acetone: toluene 1:1. Furthermore, **CHNQD-0803l** and **CHNQD-0803m** were designed with a two-step reaction to obtain chemical diversity. Furthermore, in order to explore the effect of the furan ring between the two benzene rings upon activity, three natural *p*-terphenyls, **CHNQD-0801**, **CHNQD-0802** and **CHNQD-0811**, were isolated from the same fungus (*A. candidus*). Finally, three natural products (**CHNQD-0801**, **CHNQD-0802** and **CHNQD-0811**) were obtained and thirteen *p*-terphenyl derivatives (**CHNQD-0803a–m**) were designed and synthesized by etherification and acylation reactions (Fig. 2A). The structures of these compounds were fully characterized by extensive spectroscopic methods as well as single-crystal X-ray diffraction analysis (Figs. 2B and 3). Natural products **CHNQD-0803**, **CHNQD-0801**, **CHNQD-0802**, **CHNQD-0811** and derivatives **CHNQD-0803a–m** were evaluated for their activation of AMPK in cell-based assays (Fig. 4). The results showed that all the natural products and semi-synthetic *p*-terphenyl derivatives exhibited weaker AMPK activation activity than that of **CHNQD-0803**. These results clearly indicate that 4, 3′′′ or 4′′′-hydroxyl groups are beneficial for the activity and that the furan ring between two benzene rings also has a positive effect on AMPK activation.

### CHNQD-0803 activates AMPK at cellular level

Acetyl-CoA carboxylase (ACC) is the downstream target protein of AMPK and the phosphorylation level of ACC positively correlates with the activity of AMPK. To test the effect of compounds on AMPK activity, we treated HepG2 cells with either **CHNQD-0803** or A-769662 and examined the phosphorylation level of ACC. Our results showed that **CHNQD-0803** treatment induces higher ACC phosphorylation level compared to A-769662 treatment, and this effect is dose-dependent (Supplementary Fig. S1A and B), suggesting that **CHNQD-0803** is a more potent AMPK activator.

The activation of AMPK is dependent on Thr 172 being phosphorylated by upstream Ser/Thr protein kinases, including the calcium/calmodulin-dependent protein kinase kinase-b (CaMKKβ) and the liver kinase beta 1 (LKB1) (Hawley et al. 2005; Woods et al. 2003). LKB1 is the predominant upstream kinase of AMPK in most cells (Witczak et al. 2008). To gain insight into the influence of AMPK phosphorylation level on the effects of **CHNQD-0803**, we treated non-small cell lung carcinoma H1299 cells (with normal expression level of LKB1) and A549 cells (LKB1 deficiency) (Supplementary Fig. S1C) with **CHNQD-0803**. Although **CHNQD-0803** induced AMPK activation in both cell lines in a dose-dependent manner, the activation of AMPK was more significant in H1299 cells (Supplementary Fig. S1D). The data suggest that the AMPK phosphorylation at the Thr172 site might be necessary for **CHNQD-0803**-induced activation of AMPK. Altogether, the above results demonstrate that the phosphorylation status of AMPK is necessary for the activation of AMPK by **CHNQD-0803**.

### CHNQD-0803 inhibits hepatocyte lipid accumulation

The activation of AMPK in liver can increase fatty acid oxidation and decrease fatty acid synthesis (Merrill et al. 1997; Velasco et al. 1997). In order to explore the effects of **CHNQD-0803** on lipid metabolism in liver, HepG2 cells were treated with palmitic acid (PA) to induce steatosis and then treated with **CHNQD-0803**. As expected, **CHNQD-0803** effectively reduced the lipids accumulation in PA-induced HepG2 cell (Supplementary Fig. S2A). **CHNQD-0803** decreased the level of lipid accumulation in HepG2 cells, likely through its inhibition of de novo lipogenesis indicated by the increased phosphorylation of ACC (Supplementary Fig. S2B). In the meantime, A-769662, which is a widely-used AMPK activator, also decreased lipid storage and elevated ACC phosphorylation (Supplementary Fig. S2A and B). Further, we used AMPK inhibitor Compound C to co-treat PA-induced HepG2 cells with **CHNQD-0803** and found that the compound's effect of reducing lipid accumulation disappeared (Supplementary Fig. S2C). The data suggest that **CHNQD-0803** can alleviate hepatocyte steatosis via the activation of the AMPK pathway.

Cytotoxicity is also involved in fatty acid synthesis inhibition, so colorimetric MTT assays were carried out to determine the potential cytotoxicity of the chemical series under investigation. Detection of the cytotoxicity of the **CHNQD-0803**, culminating in the consequence of **CHNQD-0803** in cancer cells (IC_50_ = 28.7 μmol/L, 26.3 μmol/L, 27.3 μmol/L in NCI-H1299, A549 and HepG2 cells, respectively. Supplementary Fig. S2D) and normal cells (IC_50_ = 71.3 μmol/L, 47.7 μmol/L in WI38 and 3T3-L1 cells, respectively. Supplementary Fig. S2E). These results suggested that **CHNQD-0803** has an extremely low toxicity in cancer cells and even less in normal cells.

### CHNQD-0803 inhibits cellular inflammation and improves hepatocyte fibrosis

Since the activation and recruitment of macrophages are important features of the inflammatory response in NASH, we choose LPS activated RAW264.7 cells as an in vitro inflammatory model to examine the anti-inflammatory effect of **CHNQD-0803** on macrophages. Macrophages were incubated with **CHNQD-0803** for 2 h, followed by stimulation with LPS for 24 h. Then, the mRNA expression level of TNF-α, IL-1β, CCL2 and iNOS were tested, and the supernatant was collected to detect the production levels of NO. The results revealed that **CHNQD-0803** suppressed pro-inflammatory cytokine mRNA expression and NO production in RAW264.7 cells (Supplementary Fig. S3A and B).

Fibrosis is an important feature of NASH development. Injured liver tissue secretes pro-fibrosis and pro-inflammatory cytokines (Pinzani and Marra 2001), in which TGF-β plays a key role in the progression of fibrosis (Meindl-Beinker and Dooley 2008). In this study, we used TGF-β to treat LX-2 cells for 12 h and then treated the cells with **CHNQD-0803** and A-769662. The results showed that **CHNQD-0803** suppressed COL1A1 expression in hepatocytes, and the effect was better than A-769662 (Supplementary Fig. S3C). In conclusion, **CHNQD-0803** effectively inhibits lipid accumulation in hepatocytes, reduces cellular inflammation, and improves hepatocyte fibrosis.

### CHNQD-0803 ameliorates hepatic steatosis in NASH mouse model

**Fig. 5:**
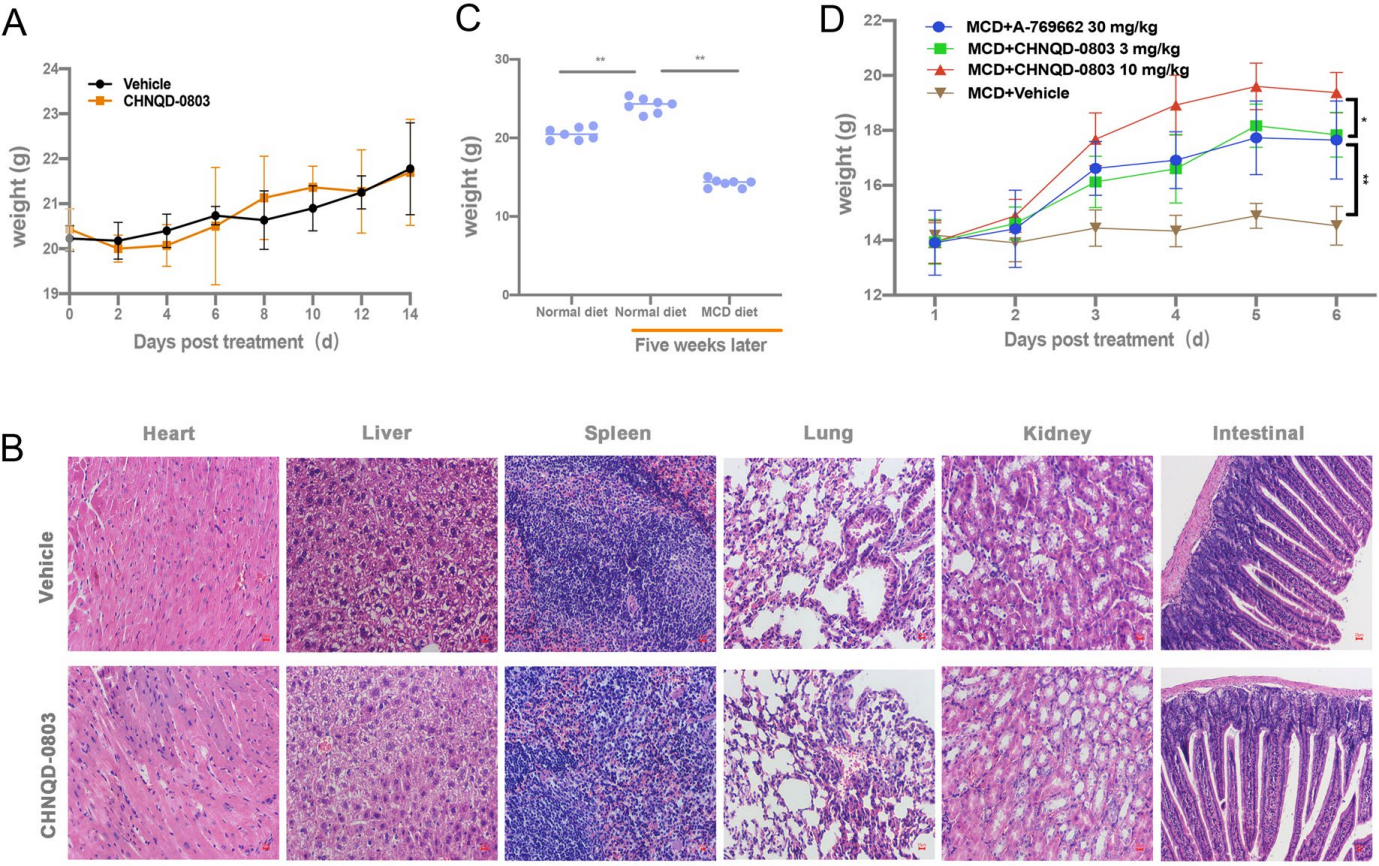
The toxicity of CHNQD-0803 and its effect on body weight in mice. **A** Effects of high-dose **CHNQD-0803** on the body weight of mice. C57BL/6 mice were injected high-dose **CHNQD-0803** (ip. 200 mg/kg) once, with body weight recorded every day after administration for two weeks. **B** Visual morphology of mice treated with high-dose **CHNQD-0803**. Hematoxylin eosin staining of heart, liver, spleen, lung, kidney and intestinal sections from representative mice. **C** Body weight of mice after MCD diet-induced. Results from at least three independent experiments. **D CHNQD-0803** ameliorated weight loss in MCD diet-induced mice. Results from at least three independent experiments. Data are expressed as mean ± SD. *P* values: ns (not significant), *P < 0.05, **P < 0.01, ***P < 0.001

To explore the safety and toxicity of **CHNQD-0803** in vivo, we performed an acute toxicity test in mice. The mice were treated with or without **CHNQD-0803** (ip. 200 mg/kg) once a day for only one day. They were then fed normally, weighed twice a week, and sacrificed after two weeks. As shown in supplementary Fig. 5A, there was no significant difference in body weight between the **CHNQD-0803** (200 mg/kg) group and the control group. Furthermore, we analyzed the H&E staining of internal organs including liver, heart, lung, spleen, kidney and intestinal tissue in control and **CHNQD-0803** (200 mg/kg) group. Results showed that **CHNQD-0803** did not significantly differ from the control group in terms of tissue morphology (Fig. 5B), indicating that there was no obvious toxicity associated with the current treatment regimen.

We used a methionine and choline deficient (MCD) dietary-induced NASH mouse model to evaluate the therapeutic impact of **CHNQD-0803** on NASH. The MCD diet feeding for 6 weeks resulted in notable weight loss (Fig. 5C). H&E staining showed inflammatory cell infiltration, hepatic steatosis and ballooning degeneration in the liver after MCD diet feeding. But these symptoms were significantly ameliorated by **CHNQD-0803** treatment, and A-769662 treatment had a similar effect (Fig. 5D, Supplementary Fig. S4A).

The basic pathological change in NASH is lipid deposition. Similarly, we also observed that the content of LDs in the MCD diet group was significantly higher than that in the normal diet group in the Oil Red O-stained mouse liver sections. This difference was evidently reduced by **CHNQD-0803** or A-769662 treatment (Supplementary Fig. S4A). Consistently, hepatic TG levels were markedly increased in the MCD diet group compared to the normal diet group, whereas **CHNQD-0803** or A-769662 treatment decreased hepatic TG levels (Supplementary Fig. S4B). The accumulation of TG in the liver resulted in reduced TG content in serum. **CHNQD-0803**, and A-769662, ameliorated the symptoms (Supplementary Fig. S4C). In summary, the data suggest that **CHNQD-0803** treatment can significantly alleviate the accumulation and deposition of lipid in hepatocytes and livers of the NASH mouse model.

### CHNQD-0803 prevents hepatic inflammation, fibrosis and liver injury in NASH mouse model

Clinically, the circulatory levels of alanine aminotransaminase (ALT) and aspartate aminotransaminase (AST) are used to assess liver damage. Therefore, we analyzed ALT and AST levels in mice serum and found that these two parameters are significantly increased in the diet-induced group compared with the normal diet group. We also found that **CHNQD-0803** or A-769662 treatment can significantly decrease serum AST and ALT levels (Supplementary Fig. S4D), indicating the protective effect of the compound on MCD-induced liver damage.

We then did immunohistochemistry staining of F4/80 positive infiltrated hepatic macrophages (Dos Anjos Cassado 2017) in liver section and found that **CHNQD-0803** treatment attenuated the MCD-induced elevation in the hepatic F4/80 positive cells. The results of RT-PCR also showed that **CHNQD-0803** treatment attenuated the MCD-induced elevation of mRNA levels of chemokine ligand (CCL2) (Supplementary Fig. S4E, F and H). In addition, **CHNQD-0803** and A-769662 also suppressed the expression of TNFα and IL-1β in MCD-induced mouse livers, two major pro-inflammatory cytokines implicated with the development of NASH (Supplementary Fig. S4H).

As NASH progresses, an uncontrolled healing response repairs damaged liver tissue, turning it into scar tissue, leading to fibrosis (Ellis and Mann 2012). The most prominent histological characteristic in the development of NASH is fibrosis. Thus, we further investigated whether **CHNQD-0803** would be able to alleviate the severity of hepatic fibrosis caused by NASH. Masson staining showed that **CHNQD-0803** significantly reduced collagen deposition in livers of NASH mice and that the effects are better than A-769662 (Supplementary Fig. S4E and G). Taken together, these results demonstrate that **CHNQD-0803** can alleviate the symptoms of NASH such as hepatic inflammation and fibrosis.

### Mechanisms of CHNQD-0803 alleviating the symptoms of NASH

Based on our findings that **CHNQD-0803** can activate AMPK in the NASH mouse model, we proposed that AMPK activation in the liver might be responsible for the therapeutic effect of **CHNQD-0803** on NASH mice. Indeed, our study shows that the phosphorylation levels of AMPK and its substrate ACC are significantly reduced in MCD diet-fed models, while **CHNQD-0803** strongly promoted their phosphorylation levels (Supplementary Fig. S5A–C). Sterol regulatory element binding protein 1c (SREBP-1c) is a key lipogenic transcription factor, which is dedicated to fatty acid uptake and triglyceride synthesis (Ikarashi et al. 2011). Increased SREBP-1c levels were found in individuals with histologically-confirmed NAFLD (Yokozawa et al. 2012). AMPK is a direct upstream kinase that can inactivate the transcription activity of SREBP-1c (Ikarashi et al. 2011). **CHNQD-0803** treatment markedly decreased the hepatic mRNA level of fatty acid synthase (FAS) and SREBP-1c (Supplementary Fig. S5D). The above results demonstrate that **CHNQD-0803** can suppress lipid synthesis in liver by inhibiting ACC and FAS.

Pro-inflammatory cytokines and chemokines are produced by injured hepatocytes. In damaged hepatocytes, NF-κB signaling is activated, resulting in expression of genes encoding TNFα and interleukin 6 (IL6), two important pro-inflammatory cytokines (Rolo et al. 2012). It appears that NF-κB and TNFα are involved in a positive feedback loop that promotes inflammation and injury to hepatocytes (Rolo et al. 2012). It is known that AMPK can inhibit NF-κB signaling (Tian et al. 2016). To further clarify the improvements in inflammation after drug treatment, we measured the expression levels of NF-κB in mouse liver tissues. We observed that **CHNQD-0803** treatment markedly attenuated the expression of hepatic NF-κB and its target gene TNFα in the livers of MCD diet-induced models (Supplementary Fig. S5E, F and Fig. S4H). Therefore, these data indicate that the AMPK-NF-κB axis is involved in **CHNQD-0803**-mediated anti-inflammation for NASH treatment.

## Discussion

NASH is a major challenge to healthcare worldwide due to the increasing global prevalence. Among patients with NASH, exercise training and dietary changes are recommended most often. However, some patients are unable to adhere to these lifestyle modifications. Thus, pharmacotherapy is an essential choice for patients with NASH. Nevertheless, there are few medications available to treat the growing burden of NASH (Chalasani et al. 2018). It is reported that AMPK modulates adipose tissue fatty acid synthesis and oxidation, attenuates hepatic steatosis, and alleviates the development of NASH (O'Neill and Hardie 2013; Scheja and Heeren 2016; Huang et al. 2015; Jian et al. 2020; Mottillo et al. 2016; Zhao et al. 2020). In this study, we identified a small molecule compound (**CHNQD-0803**) from a marine natural compound library, which showed potent therapeutic effect on NASH.

**CHNQD-0803** is originally obtained from the culture extract of *Aspergillus candidus* (Kobayashi et al. 1982), which also has been named as candidusin A. Multiple biological activities of candidusin A have been reported, including neuroprotective and antiapoptotic (Kaewin et al. 2022; Yurchenko et al. 2018). In PA-induced podocytes, candidusin A exerts cytoprotective effects by scavenging free radicals and upregulating Bcl2 (Kaewin et al. 2022). But the direct target of candidusin A has not been found. This study provided the potential mechanism for the reported biological activity of candidusin A (**CHNQD-0803**).

With regards to the mechanism by which **CHNQD-0803** activates AMPK, it may be different from two other classes of activators previously described, A-769662 and AMP. A-769662 activates AMPK by interacting with the glycogen-binding domain, while AMP interacts with the CBS motif within the γ subunit (Hardie 2007; Sanders et al. 2007). In our study, it was speculated that the binding of **CHNQD-0803** to AMPK is different from that of A-769662 based on BLI results, but its mechanism needs to be further studied.

As a polycyclic *p*-terphenyl, **CHNQD-0803** is characterized by a furan ring between the terminal and central benzene rings (Kobayashi et al. 1982). *p*-terphenyl is a type of mushroom pigment that is abundant in some edible fungi such as *Thelephora aurantiotincta* and *T. ganbajun* (Wang et al. 2014). Mushrooms are known for their antioxidant activity and the pro-apoptotic action and have become potential natural products for treating conditions such as NAFLD and obesity (Frenette et al. 2021; Huh et al. 2020). However, due to the complex composition of the mushroom, its main active ingredients and mechanisms have not been fully investigated. AMPK, as an important molecule that regulates cellular energy metabolism, maintains metabolic homeostasis through the perception of energy and glucose. It performs important functions in various metabolic syndromes, neurodegenerative diseases and even aging (Hardie and Carling 1997; Steinberg and Kemp 2009). Given the key role of AMPK in disease, perhaps **CHNQD-0803** is one of the active ingredients in the biological activity of mushrooms. The above results also provide strong support for the role of **CHNQD-0803** in the treatment of various metabolic diseases.

In conclusion, to our knowledge, this is the first time that **CHNQD-0803** has shown activity as an AMPK activator. **CHNQD-0803** effectively reduces PA-induced lipid deposition, LPS-stimulated NO production and inflammatory response, and TGF-β-induced fibrosis via activation of AMPK. These **CHNQD-0803**-mediated effects were validated in the liver of MCD-fed mice and ultimately contributed to the suppression of metabolic disorders and NASH progression. Taken together, all these findings provide solid evidence that **CHNQD-0803** is a potent and selective AMPK activator and provides a preclinical basis of the potential clinical trials for **CHNQD-0803** in NASH.

## Material and methods

### Chemistry

#### General experimental procedures

Chemicals and solvents were purchased from commercial suppliers and were used without further purification. IR spectra were obtained on a Nicolet Nexus 470 spectrophotometer in KBr discs. 1D and 2D NMR spectra were measured on a JEOL JEM-ECP NMR spectrometer (500 MHz for ^1^H and 125 MHz for ^13^C), using TMS as an internal standard. HRESIMS spectra were performed on a Thermo Scientific LTQ Orbitrap XL spectrometer. X-ray crystal data were obtained on a Bruker APEX DUO diffractometer (Cu K*α* radiation). HPLC analysis was performed on a Hitachi L-2000 system (Hitachi Ltd.) using a C18 column [(YMC Co., Ltd.) YMC-Park, ODS-A, 250 mm × 4.6 mm, S-5 μm, 12 nm, 1.0 mL/min]. Semi-preparative HPLC was performed on a Hitachi L-2000 system (Hitachi Ltd.) using a C18 column [(Eka Ltd.) Kromasil 250 mm × 10 mm, 5 μm, 2.0 mL/min]. Thin-layer chromatography (TLC) was performed on silica gel 60 F254 (Qingdao Marine Chemical Ltd.). Silica gel (Qingdao Haiyang Chemical Group Co., 200–300 mesh), octadecylsilyl silica gel (YMC Co., Ltd., 45 − 60 μm) and Sephadex LH-20 (GE Ltd.,) were used for column chromatography. Precoated silica gel plates (Yantai Zhifu Chemical Group Co., G60, F-254) were used for thin layer chromatography.

### Fungal material

The fungal strain *A. candidus* (CHNSCLM-0393) was isolated from a piece of fresh tissue from the inner part of the gorgonian coral *Juncella fragilis* (CHNNS-2016–01), collected from the Nansha Islands coral reef in the South China Sea in April 2016. The strain was identified *A. candidus* according to morphologic traits and molecular identification with the GeneBank (NCBI) accession number MF681708.

### Extraction and isolation

The organic extracts were combined and concentrated to dryness under a vacuum to obtain an EtOAc extract (926.9 g). The crude extract was fractionated into six parts (Fr.1–Fr.6) by silica gel VLC using a stepwise gradient solvent system of petroleum ether-EtOAc. Fr.2 was separated repeatedly by silica gel CC (200–300 mesh), eluting with a step gradient of petroleum ether-EtOAc from 3:1 to 0:1 (v/v) to afford five fractions (Fr.2–1 − Fr.2–4). Fr.2–2 was purified on a Sephadex LH-20 eluting with 100% MeOH, then by recrystallization to yield **CHNQD-0801** (102.7 g) and **CHNQD-0802** (20.3 g). Fr.2–3 was separated into four subfractions (Fr.2–3-1 − Fr.2–3-5) by silica gel CC (200–300 mesh), eluting with a step gradient of petroleum ether-EtOAc from 3:1 to 0:1 (v/v). Fr.2–3-1 was subjected to an ODS column eluting with 50–70% MeOH-H_2_O, then applied on semipreparative HPLC eluting with 40–60% MeOH-H_2_O to yield pure **CHNQD-0811** (42.3 mg). Fr.2–3-2 was further purified by Sephadex LH-20 CC with 100% MeOH and recrystallization to give **CHNQD-0803** (7.0 g).

**CHNQD-0803**: gray powder; ^1^H NMR (500 MHz, DMSO-*d*_6_) *δ* 9.53 (1H, br. s), 9.41 (1H, br. s), 9.10 (1H, br. s), 7.42 (2H, d, *J* = 8.5 Hz), 7.36 (1H, s), 7.07 (1H, s), 6.85 (2H, d, *J* = 8.5 Hz), 6.71 (1H, s), 3.97 (3H, s), 3.75 (3H, s); ^13^C NMR (125 MHz, DMSO-*d*_6_) *δ* 156.7 (C), 149.4 (C × 2), 148.4 (C), 145.8 (C), 142.5 (C), 135.9 (CH),130.5 (C), 130.4 (CH × 2), 128.6 (C), 115.1 (CH × 2), 114.0 (C), 113.6 (C), 107.0 (CH), 105.6 (CH), 98.5 (CH), 60.6 (CH_3_), 55.8 (CH_3_).

**CHNQD-0811**: gray powder; ^1^H NMR (400 MHz, DMSO-*d*_6_) *δ* 9.44 (1H, br. s), 9.18 (1H, br. s), 7.59 (2H, d, *J* = 7.2 Hz), 7.46 (2H, t, *J* = 7.5 Hz), 7.39 (1H, m), 7.37 (1H, s), 7.09 (1H, s), 6.77 (1H, s), 3.99 (3H, s), 3.79 (3H, s); ^13^C NMR (100 MHz, DMSO-*d*_6_) *δ* 149.5 (C), 149.4 (C), 148.2 (C), 146.1 (C), 142.6 (C), 138.1 (C), 136.1 (CH), 130.3 (C), 129.3 (CH × 2), 128.2 (CH × 2), 127.1 (CH), 114.8 (C), 113.5 (C), 107.1 (CH), 105.8 (CH), 98.5 (CH), 60.7 (CH_3_), 55.9 (CH_3_).

**CHNQD-0801**: white powder; ^1^H NMR (400 MHz, DMSO-*d*_6_) *δ* 9.51 (1H, br. s), 9.30 (1H, br. s), 8.50 (1H, br. s), 7.44 (2H, d, *J* = 8.6 Hz), 7.10 (2H, d, *J* = 8.6 Hz), 6.84 (2H, d, *J* = 8.6 Hz), 6.75 (2H, d, *J* = 8.6 Hz), 6.39 (1H, s), 3.64 (3H, s), 3.30 (3H, s); ^13^C NMR (100 MHz, DMSO-*d*_6_) *δ* 156.7 (C), 155.9 (C), 153.1 (C), 148.1 (C), 139.3 (C), 132.3 (C), 131.8 (CH × 2), 129.7 (CH × 2), 128.7 (C), 124.5 (C), 116.9 (C), 115.2 (CH × 2), 114.3 (CH × 2), 103.0 (CH), 60.0 (CH_3_), 55.6 (CH_3_).

**CHNQD-0802**: white powder; ^1^H NMR (400 MHz, DMSO-*d*_6_) *δ* 9.51 (1H, br. s), 8.74 (2H, br. s), 8.43 (1H, br. s), 7.44 (1H, d, *J* = 8.6 Hz), 6.84 (1H, d, *J* = 8.6 Hz), 6.71 (1H, d, *J* = 8.1 Hz), 6.69 (1H, d, *J* = 2.0 Hz), 6.55 (1H, dd, *J* = 8.1, 2.0 Hz), 6.38 (1H, s), 3.63 (3H, s), 3.30 (3H, s); ^13^C NMR (100 MHz, DMSO-*d*_6_) *δ* 156.7 (C), 153.1 (C), 148.1 (C), 144.3 (C), 143.9 (C), 139.3 (C), 132.2 (C), 129.7 (CH × 2), 128.8 (C), 125.0 (C), 121.9 (CH), 118.4 (CH), 117.3 (C), 115.2 (CH × 2), 114.8 (CH), 102.9 (CH), 60.0 (CH_3_), 55.6 (CH_3_).

### General synthetic methods for CHNQD-0803a–j

To a stirred solution of **CHNQD-0803** (1 equiv) in dry acetone (20 mL) were added the iodomethane, 3-bromopropene or acetyl chloride (6–10 equiv) and anhydrous K_2_CO_3_. The reaction mixture was stirred at 45 °C (rt. for acetyl chloride) for 4–8 h and then quenched with water and diluted with ethyl acetate. The organic layer was separated and the organic solvents were removed under a vacuum. The residue was purified by silica gel CC followed by semipreparative HPLC to yield **CHNQD-0803a–j**.

**CHNQD-0803a**: gray powder; ^1^H NMR (400 MHz, DMSO-*d*_6_) *δ* 9.59 (1H, br. s), 9.06 (1H, brs), 7.42 (2H, d, *J* = 8.5 Hz), 7.40 (1H, s), 7.38 (1H, s), 6.85 (2H, d, *J* = 8.5 Hz), 6.73 (1H, s), 3.98 (3H, s), 3.87 (3H, s), 3.77 (3H, s); ^13^C NMR (100 MHz, DMSO-*d*_6_) *δ* 156.8 (C), 149.6 (C), 149.3 (C), 148.6 (C), 148.0 (C), 143.6 (C), 136.0 (C), 131.0 (C), 130.4 (CH × 2), 128.6 (C), 115.1 (CH × 2), 114.6 (C), 113.8 (C), 106.9 (CH), 105.7 (CH), 96.2 (CH), 60.6 (CH_3_), 56.1 (CH_3_), 55.9 (CH_3_). HRESIMS *m/z* 367.1171 [M + H]^+^ (calcd for C_21_H_19_O_6_^+^, 367.1176).

**CHNQD-0803b**: white powder; ^1^H NMR (400 MHz, DMSO-*d*_6_) *δ* 9.62 (1H,brs), 7.46 (1H, s), 7.44 (4H,m), 6.86 (2H, d, *J* = 8.6 Hz), 6.76 (1H, s), 4.00 (3H, s), 3.87 (3H, s), 3.86 (3H, s), 3.78 (3H, s); ^13^C NMR (100 MHz, DMSO-*d*_6_) *δ* 156.8 (C), 150.0 (C), 149.6 (C), 149.2 (C), 148.6 (C), 146.1 (C), 136.0 (C), 131.1 (C), 130.4 (CH × 2), 128.5 (C), 115.2 (CH × 2), 114.2 (C), 113.8 (C), 105.8 (CH), 104.0 (CH), 96.3 (CH), 60.6 (CH_3_), 56.1 (CH_3_), 56.0 (CH_3_), 55.9 (CH_3_). HRESIMS *m/z* 381.1323 [M + H]^+^ (calcd for C_22_H_21_O_6_^+^, 381.1333).

**CHNQD-0803c**: white powder; ^1^H NMR (400 MHz, DMSO-*d*_6_) *δ* 9.07 (1H, brs), 7.54 (2H, d, *J* = 8.7 Hz), 7.40 (1H, s), 7.39 (1H, s), 7.03 (2H, d, *J* = 8.7 Hz), 6.75 (1H, s), 3.99 (3H, s), 3.87 (3H, s), 3.81 (3H, s), 3.79 (3H, s); ^13^C NMR (100 MHz, DMSO-*d*_6_) *δ* 158.5 (C), 149.6 (C), 149.3 (C), 148.5 (C), 148.1 (C), 143.6 (C), 136.0 (C), 130.5 (C), 130.4 (CH × 2), 130.2 (C), 114.5 (C), 114.1 (C), 113.7 (CH × 2), 106.9 (CH), 105.7 (CH), 96.2 (CH), 60.6 (CH_3_), 56.1 (CH_3_), 55.9 (CH_3_), 55.2 (CH_3_). HRESIMS *m/z* 381.1325 [M + H]^+^ (calcd for C_22_H_21_O_6_^+^, 381.1333).

**CHNQD-0803d**: white powder; ^1^H NMR (400 MHz, DMSO-*d*_6_) *δ* 7.55 (2H, d, *J* = 8.8 Hz), 7.47 (1H, s), 7.46 (1H, s), 7.03 (1H, d, *J* = 8.8 Hz), 6.79 (1H, s), 4.01 (3H, s), 3.87 (3H, s), 3.86 (3H, s), 3.81 (3H, s), 3.80 (3H, s); ^13^C NMR (100 MHz, DMSO-*d*_6_) *δ* 158.5 (C), 150.0 (C), 149.6 (C), 149.3 (C), 148.5 (C), 146.1 (C), 136.0 (C), 130.6 (C), 130.4 (CH × 2), 130.1 (C), 114.1 (C), 114.0 (C), 113.7 (CH × 2), 105.8 (CH), 104.0 (CH), 96.3 (CH), 60.6 (CH_3_), 56.1 (CH_3_), 56.0 (CH_3_), 55.9 (CH_3_), 55.1 (CH_3_). HRESIMS *m/z* 395.1481 [M + H]^+^ (calcd for C_23_H_23_O_6_^+^, 395.1489).

**CHNQD-0803e**: gray powder; ^1^H NMR (400 MHz, DMSO-*d*_6_) *δ* 9.57 (1H, s), 9.06 (1H, s), 7.42 (2H, d, *J* = 8.6 Hz), 7.42 (1H, s), 7.37 (1H, s), 6.85 (2H, d, *J* = 8.6 Hz), 6.73 (1H, s), 6.10 (1H, m), 5.48 (1H, dd, *J* = 17.3, 1.8 Hz), 5.29 (1H, dd, *J* = 10.5, 1.6 Hz), 4.67 (2H, d, *J* = 5.3 Hz), 3.98 (3H, s), 3.76 (3H, s); ^13^C NMR (100 MHz, DMSO-*d*_6_) *δ* 156.8 (C), 149.6 (C), 149.1 (C), 148.7 (C), 146.7 (C), 143.8 (C), 136.0 (C), 133.8 (CH_2_), 131.1 (C), 130.4 (CH × 2), 128.5 (C), 117.7 (CH), 115.1 (CH × 2), 114.9 (C), 113.7 (C), 107.2 (CH), 105.7 (CH), 97.7 (CH), 69.4 (CH_2_), 60.6 (CH_3_), 55.9 (CH_3_). HRESIMS *m/z* 393.1324 [M + H]^+^ (calcd for C_23_H_21_O_6_^+^, 393.1333).

**CHNQD-0803f**: white powder; ^1^H NMR (400 MHz, DMSO-*d*_6_) *δ* 9.56 (1H, s), 7.51 (1H, s), 7.46 (1H, s), 7.44 (2H, d, *J* = 8.5 Hz), 6.86 (2H, d, *J* = 8.6 Hz), 6.76 (1H, s), 6.11 (2H, m), 5.49 (1H, dd, *J* = 6.5, 1.8 Hz), 5.45 (1H, dd, *J* = 6.6, 1.8 Hz), 5.31 (1H, dd, *J* = 6.3, 1.7 Hz), 5.28 (1H, m), 4.69 (2H, d, *J* = 5.3 Hz), 4.64 (2H, d, *J* = 5.3, Hz), 4.00 (4H, s), 3.77 (4H, s); ^13^C NMR (100 MHz, DMSO-*d*_6_) *δ* 156.8 (C), 150.2 (C), 149.6 (C), 148.7 (C), 148.4 (C), 145.1 (C), 135.9 (C), 134.0 (CH_2_), 133.6 (CH_2_), 131.3 (C), 130.4 (CH × 2), 128.4 (C), 117.6 (CH), 117.4 (CH), 115.1 (CH × 2), 114.6 (C), 113.6 (C), 106.7 (CH), 105.8 (CH), 97.9 (CH), 70.0 (CH_2_), 69.3 (CH_2_), 60.6 (CH_3_), 55.9 (CH_3_). HRESIMS *m/z* 433.1633 [M + H]^+^ (calcd for C_26_H_25_O_6_^+^, 433.1646).

**CHNQD-0803g**: white powder; ^1^H NMR (400 MHz, acetone-*d*_6_) *δ* 7.57 (2H, d, *J* = 8.8 Hz), 7.53 (1H, s), 7.32 (1H, s), 7.04 (2H, d, *J* = 8.8 Hz), 6.79 (1H, s), 6.14 (2H, m), 5.49 (2H, m), 5.29 (2H, m), 4.77 (2H, d, *J* = 5.2 Hz), 4.64 (2H, d, *J* = 5.2 Hz), 4.06 (3H, s), 3.84 (3H, s); ^13^C NMR (100 MHz, acetone-*d*_6_) *δ* 158.0 (C), 150.2 (C), 149.8 (C), 149.3 (C), 146.3 (C), 143.7 (C), 136.5 (C), 133.9 (CH_2_), 133.5 (CH_2_), 131.3 (C), 131.0 (C), 130.5 (CH × 2), 117.2 (CH), 116.5 (CH), 115.8 (C), 114.6 (C), 114.3 (CH × 2), 107.2 (CH), 105.6 (CH), 96.7 (CH), 70.0 (CH_2_), 68.4 (CH_2_), 60.2 (CH_3_), 55.4 (CH_3_). HRESIMS *m/z* 433.1644 [M + H]^+^ (calcd for C_26_H_25_O_6_^+^, 433.1646).

**CHNQD-0803h**: white powder; ^1^H NMR (400 MHz, DMSO-*d*_6_) *δ* 7.55 (1H, s), 7.53 (2H, d, *J* = 8.8 Hz), 7.47 (1H, s), 7.05 (2H, d, *J* = 8.8 Hz), 6.79 (1H, s), 6.10 (3H, m), 5.38 (6H, m), 4.69 (2H, d, *J* = 5.2 Hz), 4.64 (4H, m), 4.01 (3H, s), 3.79 (3H, s);; ^13^C NMR (100 MHz, DMSO-*d*_6_) *δ* 157.5 (C), 150.2 (C), 149.6 (C), 148.6 (C), 148.4 (C), 145.1 (C), 136.0 (C), 134.0 (CH_2_), 133.8 (CH_2_), 133.5 (CH_2_), 130.7 (C), 130.4 (CH × 2), 130.2 (C), 117.6 (CH), 117.5 (CH), 117.3 (CH), 114.5 (C), 114.5 (CH × 2), 113.9 (C), 106.7 (CH), 105.9 (CH), 97.9 (CH), 70.0 (CH_2_), 69.3 (CH_2_), 68.2 (CH_2_), 60.7 (CH_3_), 55.9 (CH_3_). HRESIMS *m/z* 473.1947 [M + H]^+^ (calcd for C_29_H_29_O_6_^+^, 473.1959).

**CHNQD-0803i**: gray powder; ^1^H NMR (400 MHz, DMSO-*d*_6_) *δ* 9.61 (1H, s), 7.83 (1H, s), 7.77 (1H, s), 7.47 (2H, d, *J* = 8.6 Hz), 6.87 (2H, d, *J* = 8.6 Hz), 6.85 (1H, s), 4.02 (3H, s), 3.78 (3H, s), 2.33 (6H, s); ^13^C NMR (100 MHz, DMSO-*d*_6_) *δ* 168.8 (C), 168.5 (C), 157.1 (C), 152.1 (C), 150.1 (C), 149.6 (C), 140.8 (C), 138.5 (C), 135.9 (C), 133.5 (C), 130.5 (CH × 2), 128.1 (C), 120.7 (C), 115.9 (C), 115.2 (CH × 2), 112.3 (CH), 107.2 (CH), 106.5 (CH), 60.8 (CH_3_), 56.1 (CH_3_), 20.5 (CH_3_), 20.4 (CH_3_). HRESIMS *m/z* 437.1215 [M + H]^+^ (calcd for C_24_H_21_O_8_^+^, 437.1231).

**CHNQD-0803j**: white powder; ^1^H NMR (400 MHz, DMSO-*d*_6_) *δ* 7.87 (1H, s), 7.79 (1H, s), 7.67 (2H, d, *J* = 8.2 Hz), 7.24 (2H, d, *J* = 8.2 Hz), 6.92 (1H, s), 4.04 (3H, s), 3.86 (3H, s), 2.34 (6H, s), 2.31 (3H, s); ^13^C NMR (100 MHz, DMSO-*d*_6_) *δ* 169.3 (C), 168.7 (C), 168.4 (C), 152.2 (C), 150.1 (C), 149.9 (C), 149.3 (C), 141.0 (C), 138.6 (C), 136.0 (C), 135.0 (C), 132.3 (C), 130.5 (CH × 2), 121.7 (CH × 2), 120.5 (C), 116.1 (C), 113.2 (CH), 107.3 (CH), 106.7 (CH), 61.0 (CH_3_), 56.2 (CH_3_), 20.9 (CH_3_), 20.4 (CH_3_ × 2). HRESIMS *m/z* 501.1138 [M + Na]^+^ (calcd for C_26_H_22_O_9_Na^+^, 501.1156).

### General synthetic methods for CHNQD-0803k

The **CHNQD-0803** (1 equiv) and corresponding P_2_O_5_ (40 equiv) were added in acetone: toluene 1:1 (10 mL) and then the reaction mixture was stirred at 90 °C for 5–8 h and then quenched with saturated NaHCO_3_ solution and diluted with EtOAc. The EtOAc layer was separated and dried under vacuum. Then the crude residue was purified through silica gel column chromatography followed by semipreparative HPLC to yield **CHNQD-0803k**.

**CHNQD-0803k**: yellow oil; ^1^H NMR (400 MHz, DMSO-*d*_6_) *δ* 9.54 (1H, brs), 7.43 (2H, d, *J* = 8.7 Hz), 7.33 (1H, s), 7.32 (1H, s), 6.85 (2H, d, *J* = 8.6 Hz), 6.74 (1H, s), 3.98 (3H, s), 3.76 (3H, s), 1.70 (6H, s); ^13^C NMR (100 MHz, DMSO-*d*_6_) *δ* 156.8 (C), 150.3 (C), 149.5 (C), 148.7 (C), 146.7 (C), 144.0 (C), 135.9 (C), 131.0 (C), 130.4 (CH × 2), 128.4 (C), 119.0 (C), 115.1 (CH × 2), 115.1 (C), 113.8 (C), 105.8 (CH), 100.4 (CH), 94.0 (CH), 60.6 (CH_3_), 55.9 (CH_3_), 25.5 (CH_3_ × 2). HRESIMS *m/z* 393.1319 [M + H]^+^ (calcd for C_23_H_21_O_6_^+^, 393.1333).

### General synthetic methods for CHNQD-0803l–m

To a stirred solution of **CHNQD-0803k** (1 equiv) in dry acetone (20 mL) were added the iodomethane or acetyl chloride (3–6 equiv) and anhydrous K_2_CO_3_. The reaction mixture was stirred at 45 °C (rt. for acetyl chloride) for 4–8 h and then quenched with water and diluted with ethyl acetate. The organic layer was separated and the organic solvents were removed under a vacuum. The residue was purified by silica gel CC followed by semipreparative HPLC to yield **CHNQD-0803l–m**.

**CHNQD-0803l**: white powder; ^1^H NMR (400 MHz, DMSO-*d*_6_) *δ* 7.54 (1H, d, *J* = 8.8 Hz), 7.34 (1H, s), 7.33 (1H, s), 7.03 (2H, d, *J* = 8.8 Hz), 6.76 (1H, s), 3.99 (3H, s), 3.81 (3H, s), 3.77 (3H, s), 1.70 (6H, s); ^13^C NMR (100 MHz, DMSO-*d*_6_) *δ* 158.6 (C), 150.4 (C), 149.6 (C), 148.7 (C), 146.9 (C), 144.1 (C), 136.0 (C), 130.6 (C), 130.5 (CH × 2), 130.1 (C), 119.1 (C), 115.1 (C), 114.1 (C), 113.8 (CH × 2), 105.9 (CH), 100.5 (CH), 94.1 (CH), 60.8 (CH_3_), 56.0 (CH_3_), 55.2 (CH_3_), 25.5 (CH_3_ × 2). HRESIMS *m/z* 407.1483 [M + H]^+^ (calcd for C_24_H_23_O_6_^+^, 407.1489).

**CHNQD-0803m**: white powder; ^1^H NMR (400 MHz, DMSO-*d*_6_) *δ* 7.63 (2H, d, *J* = 8.2 Hz), 7.36 (1H, s), 7.34 (1H, s), 7.22 (2H, d, *J* = 8.2 Hz), 6.82 (1H, s), 4.00 (3H, s), 3.83 (3H, s), 2.31 (3H, s), 1.70 (6H, s); ^13^C NMR (100 MHz, DMSO-*d*_6_) *δ* 169.3 (C), 150.4 (C), 149.7 (C), 149.6 (C), 148.4 (C), 147.0 (C), 144.1 (C), 136.0 (C), 135.4 (C), 130.4 (CH × 2), 129.8 (C), 121.7 (CH × 2), 119.1 (C), 115.0 (C), 114.7 (C), 106.0 (CH), 100.5 (CH), 94.0 (CH), 60.8 (CH_3_), 56.1 (CH_3_), 25.5 (CH_3_ × 2), 20.9 (CH_3_). HRESIMS *m/z* 435.1427 [M + H]^+^ (calcd for C_25_H_23_O_7_^+^, 435.1438).

### X-ray crystallographic analysis of CHNQD-0803, 0803c and 0803d

Colorless crystals of **CHNQD-0803**, **0803c** and **0803d** suitable for X-ray diffraction were obtained from MeOH by slow evaporation. The crystal data were collected at 293 K on an Agilent Gemini Ultra diffractometer with Cu K*α* radiation (*λ* = 1.54184 Å). The structure was solved by direct methods (SHELXS-97) and refined using full-matrix least-squares difference Fourier techniques. All non-hydrogen atoms were refined anisotropically. The crystallographic data for **CHNQD-0803**, **0803c** and **0803d** have been deposited at the Cambridge Crystallographic Data Centre with the deposition numbers 2144258, 2,144,264 and 2,144,262. These data can be obtained, free of charge, on application to the Director, CCDC, 12 Union Road, Cambridge CB21EZ, UK.

*Crystal data for* **CHNQD-0803**: C_20_H_16_O_6_, *M*r = 335.31, monoclinic, space group *P* 21/*n* with *a* = 17.3700(10) Å, *b* = 7.0047(5) Å, *c* = 21.1623(14), *α* = *γ* = 90°, *β* = 109.080(6), *V* = 2433.4(3) Å^3^, *Z* = 4, *D*x = 1.279 mg/m^3^,* μ* (Cu K*α*) = 0.787 mm^−1^, and *F* (000) = 992. Crystal dimensions: 0.08 × 0.08 × 0.07 mm^3^. Independent reflections: 4351 (*R*_int_ = 0.0514). The final *R*_1_ values was 0.0389.

*Crystal data for* **CHNQD-0803c**: C_22_H_20_O_6_, *M*r = 380.38, triclinic, space group *P*-1 with *a* = 7.4648(11) Å, *b* = 9.7303(12) Å, *c* = 13.0580(18) Å, *α* = 85.969(11), *β* = 77.632(12), *γ* = 86.994(11), *V* = 923.5(2) Å^3^, *Z* = 2, *D*x = 1.368 mg/m^3^, *μ* (Cu K*α*) = 0.826 mm^−1^, and *F* (000) = 400. Crystal dimensions: 0.12 × 0.12 × 0.11 mm^3^. Independent reflections: 3285 (*R*_int_ = 0.0691). The final *R*_1_ value was 0.0448.

*Crystal data for* **CHNQD-0803d**: C_23_H_22_O_6_, *M*r = 394.42, monoclinic, space group *P* 21 with *a* = 10.2568(3) Å, *b* = 7.14832(19) Å, *c* = 26.7537(8) Å, *α* = *γ* = 90°, *β* = 99.459(3), *V* = 1934.87(9) Å^3^, *Z* = 2, *D*x = 1.354 mg/m^3^, *μ* (Cu K*α*) = 0.808 mm^−1^, and *F* (000) = 832. Crystal dimensions: 0.08 × 0.08 × 0.07 mm^3^. Independent reflections: 6291 (*R*_int_ = 0.0461). The final *R*_1_ value was 0.0323.

### Animal experiments

C57BL/6 J mice (Male, 8 weeks old) were provided by the Charles River Experimental Animal Co., Ltd. (Beijing, China). Mice were acclimatized for at least 1 week and then randomly assigned to five groups: One group was fed with a standard chow diet and others group were fed MCD. After 6 weeks, MCD groups for the 7 days treatment were as follows: vehicle (5% DMSO, 10% Solutol, 85% Saline, i.p., b.i.d.), **CHNQD-0803** (3 or 10 mg/kg, i.p., b.i.d.) or 10 mg/kg of A-769662 (i.p., b.i.d.). All animals received i.p. injections twice daily. ALT and AST were determined in final blood samples taken at the end of the 7-day research. Total cholesterol, total triglycerides, and HDL levels were also determined. Moreover, the liver tissues were taken and promptly frozen in liquid nitrogen for future investigation and testing.

### Molecular reagents

Dulbecco’s modified Eagle’s medium (DMEM) and Roswell Park Memorial Institute (RPMI) 1640 were provided by GIBCO (Grand Island, USA). Fetal bovine serum (FBS) was from PAN (Germany). Streptomycin and penicillin were provided by Hyclone (Hangzhou, China). MTT, palmitic acid (PA), and DMSO were provided from Sigma (St. Louis, USA). Cellular and liver TG detection kit were purchased from Solarbio (Beijing, China). Blood biochemical analysis kits for aminotransferase (ALT), alanine aspartate aminotransferase (AST), high-density lipoprotein (HDL-C), total cholesterol (TC) and triglyceride (TG) were from DEWEI (Foshan, China). RIPA, PMSF, BCA kit and Oil Red O kit were from Yamei (Shanghai, China). Enhanced chemiluminescence liquid (ECL) was from Sparkjade (Shandong, China) and TGF-β1 from Peprotech (Rocky Hill, USA). PVDF membranes were products of Millipore (USA). AMPKα (cat# 2532S), pAMPK (Thr172, cat# 2535S), pACC (Ser79, cat# 11818S), COL1A1 (cat# 72026S), F4/80 (cat# 70,076) and NF-κB (cat# 8242), antibodies were provided by Cell Signaling Technology (Boston, USA). ACC (cat# 67,373–1-Ig), β-Tubulin (cat# 10,094–1-AP) and β-Actin (cat# 66,009–1-Ig) antibodies were provided by ProteinTech (Wuhan, China). Secondary antibodies against primary antibodies were from Millipore (Darmstadt, Germany). Compound C and A-769662 were from Selleck Chemicals (Houston, Texas, USA). TRIzol reagent was from Thermo (Thermo, Waltham, United States). SYBR Green Master was provided by Roche (Mannheim, Germany). PrimeScript RT reagent kit, B*am*H I and S*ph* I were from Takara (Dalian, China). Nitric Oxide Assay Kit was from UElandy (Suzhou, China). KCl, NaCl, MgCl_2_, HCl and other chemical reagents were from Sinopharm (Shanghai, China).

### Cell culture and reagents

Human lung cancer cell lines A549 and H1299 were from Shanghai Cell Bank (Shanghai, China), Chinese Academy of Science. A549 and H1299 were cultured in RPMI-1640. RAW264.7, LX-2, HepG2, WI38 and 3T3-L1 cells were from Shanghai Cell Bank (Shanghai, China), Chinese Academy of Science. RAW264.7, LX-2, HepG2, WI38 and 3T3-L1 cells were cultured in DMEM. The above-mentioned medium contained 10% FBS, penicillin (107 U/L) and streptomycin (10 mg/L) at 37 °C with 5% CO_2_.

### MTT viability assay

**CHNQD-0803** was added to 96-well plates that have been pre-seeded with cells at a concentration range of 0–200 μmol/L. Fresh DMSO and sterile distilled water were used as controls for normal growth and non-specific color, respectively. After 48 h of treatment, medium was discarded from each well. Cells were washed three times before being used. Incubation the proceeded at 37 °C for 4 h with freshly produced MTT solution in clear medium (1 mg/mL) followed by washing. DMSO was used to dissolve the formazan crystals after removing MTT. An absorbance measurement at 570 nm was then performed on a microplate reader (Molecular Devices, CA, USA).

### Construct of AMPK expression vector

Human AMPK subunits (flag-α1, AVI-β1, His-γ1) were cloned into a pET28a vector. The nucleic acid sequence was designed according to the cDNA sequence of AMPK *α*1, *β*1, *γ*1 3 subunits (GenBank Accession Nos. X95578, X95577 and U40819) and optimized using *E. coli* preferred codons, with *Bam*H I and *Sph* I sites added at the ends of the sequence. The DNA fragment containing the target gene was synthesized by Nanjing Kinco Bioengineering Technology Service Co., Ltd. The synthesized AMPK α1, β1, and γ1 3 subunit gene fragments were double-enzyme digested with *Bam*H I and *Sph* I and then ligated to the pET28a that was digested with the same enzymes. The new vector was called pET28a-AMPK.

### Expression and purification of recombinant AMPK

The plasmid pET28a-AMPK was transfected into competent cells (*E. coli* BL21 expressing CAMKKβ) and grown overnight at 37 °C containing 50 mg/mL kanamycin and 100 mg/mL ampicillin. Inoculating LB medium with antibiotics using resuspended cells was performed. Cultures were placed at 37 °C in a shaker and grown for about 2 h until the concentration reached 0.4–0.6 OD_600_ nm, then induced with 0.5 mmol/L isopropyl β-D-thiogalactopyranoside (IPTG). The culture was transferred to 20 °C for an additional 24 h. After harvesting the cells, they were washed with PBS, resuspended in lysis buffer (50 mmol/L NaCl, 50 mmol/L Na-phosphate, 1 mmol/L β-mercaptoethanol and 10 mmol/L imidazole, pH 8.0), and sonicated on ice (30% duty, 10 min, two times). A centrifuge was used to remove the insoluble material, and the supernatant was loaded onto Ni–NTA agarose. The agarose was washed with the lysis buffer containing 20 mmol/L imidazole (three column volumes) and eluted with 250 mmol/L imidazole (lysis buffer). The protein was then stored at −80 °C.

### AMPK kinase assays

In a 60 μL reaction volume, 20 μmol/L SAMS peptide, 2 mol/L ATP, AMPK, and compounds at the indicated concentrations were incubated at 30 °C for 15 min in a buffer (40 mmol/L HEPES, 5 mmol/L MgCl_2_, 80 mmol/L NaCl, 1 mmol/L DTT, 0.8 mmol/L EDTA, 8% glycerol and 0.18% Triton-X-100). Guanidine HCl (7.5 mol/L) was added to stop the reaction, 30 μL of which was then transferred to the final reaction mixture in a 96-well plate pre-coated with streptavidin to react at room temperature for 20 min, wash with PBST and add pACC (Ser79) antibody, incubate at 37 °C for 1 h and add secondary antibody. TMB was added after washing three times with PBST and placed in the dark for 0.5 h at 37 °C for color reaction. A microplate reader was used to measure the absorbance at OD_450_ nm.

### Biolayer interferometry binding assays (BLI)

With the use of an Octet-Red 96 (Pall-ForteBio), BLI was used to examine the competition assay of **CHNQD-0803** and A-769662 (Pall-ForteBio). It is necessary to activate the NTA biosensors by incubating them in phosphate-buffered saline with Tween 20 (pH 7.2, PBST). The tests consisted of the following processes performed at 30 °C: equilibration (60 s), immobilization of AMPK protein (80 μg/mL) onto sensors (600 s), equilibration (120 s), loading of the first molecule (180 s), loading of the second molecule in the presence of the first molecule (180 s), dissociation of molecule for measurement (180 s).

### Western blotting

Cells or liver tissue were lysed in protein lysis buffer, which included phosphatase inhibitors and a cocktail of protease inhibitors. A BCA kit was used to determine the total protein concentration. After protein denaturation, SDS-PAGE electrophoresis was performed and the protein transferred from the gel to a PVDF membrane. After transfer, the membrane was incubated with 5% nonfat milk and blocked for 1 h at room temperature. Primary antibodies were incubated overnight at 4 °C, washed three times with TBST, and membranes were incubated with secondary antibodies dissolved in 5% nonfat milk. The signals were detected using ECL chemiluminescence. Quantifying the relative intensities was done using Image Lab (Tanon, China). Image J was used to determine band intensities.

### Biochemical assay

In order to extract serum, blood samples were taken in noncoated tubes. The serum was produced by centrifuging at 3000 r/min for 20 min at room temperature to get a concentrated suspension. Before analysis, samples were transferred to polypropylene micro tubes and kept at − 80 °C until needed. An automated biochemical analyzer (Mindray, China) was used to evaluate the alanine aminotransferase (ALT) and aspartate aminotransferase (AST) contents in the blood samples after they had been thawed at 4 °C for 30 min.

### Histological analysis (H&E) and sirius red analysis

After removal of mouse liver tissue, it was quickly fixed in 4% paraformaldehyde. Tissues were dehydrated in ethanol, paraffin embedded, sectioned, and stained with Sirius red and hematoxylin–eosin (H&E) using standard protocols. Images were photographed using an optical microscope (Olympus, Tokyo, Japan).

### Oil red O (ORO) staining

Growth media was removed and cells fixed with 4% paraformaldehyde for 30 min at room temperature after washing. The cells were stained with diluted oil red O solution after they had been fixed. The dye solution was dumped after staining, and the excess dye solution was cleaned. After the stained cells were photographed, they were decolorized with isopropanol and absorbance at 490 nm was detected. For histological study, liver tissue sections of mice were immersed in frozen section medium and stored at − 20 °C. The desired liver tissue section was then removed, placed on a slide, air dried for 1 day, and stained with Oil Red O.

### Immunohistochemistry

Liver tissue was removed from mice and was rapidly immersed in 4% paraformaldehyde. The paraformaldehyde-fixed liver tissue was embedded, sliced, deparaffinized, and antigen recovered. Antibodies against F4/80 were incubated after sections were blocked with non-immunoreactive serum. Incubation with goat anti-rabbit was followed by the addition of diaminobenzidine (DAB) chromogen and hematoxylin counterstaining. The sections were observed and analyzed using an optical microscope.

### RNA extraction and reverse transcription polymerase chain reaction (RT-PCR)

Following the manufacturer’s instructions, RNA from mouse liver tissue was extracted using the TRIzol reagent. In order to acquire cDNA, a reverse transcription kit was used to carry out RT-PCR according to the instrument’s specifications. The specific primers were synthesized by Tsingke, and primer sequences can be found in the supporting information.

### Surface-plasmon resonance (SPR)

The Biacore T200 System (GE Healthcare) was used to analyze the interaction between chemicals and AMPK proteins and to determine their kinetic constants. Sulpho-NHS/EDC chemistry was used to activate the CM5 Sensor Chip in a buffer (pH 7.4, 137 mmol/L NaCl, 2.7 mmol/L KCl, and 0.05 percent (v/v) surfactant P20). The chip was immobilized with 50 g/mL recombinant human AMPK protein in sodium acetate, pH 4.5, and then blocked with 1 mol/L ethanolamine, pH 8.0. Ligands were dissolved to a concentration of 10 mmol/L in 100 percent DMSO and subsequently 200-fold diluted in running buffer without DMSO to 50, 25, 12.5, 6.25, 3.125, 1.56, 0.78, and 0 mmol/L before injection. The optical interference pattern was quantified in terms of a change in the optical path difference in nm. Biacore T200 Evaluation Software was used to examine the data.

### Statistics analysis and drawing

Data was presented as mean ± SD and analyzed using 2-tailed t test or 1-way analysis of variance with a Tukey’s post hoc test. When the *P* value was < 0.05, the difference was termed as significant.

## Supplementary Information

Below is the link to the electronic supplementary material.

Supplementary file1 (DOCX 6657 KB)

## Data Availability

The data that supports the findings of this study are included in this published article (and its supplementary information file).
