# Supplementary material for: Discovery of a natural small-molecule AMP-activated kinase activator that alleviates nonalcoholic steatohepatitis
Source: Mar Life Sci Technol. 2023 Apr 30;5(2):196–210. doi: 10.1007/s42995-023-00168-z (PMC10232707; doi:10.1007/s42995-023-00168-z)
Supplement: Supplementary file 1 — Supplementary file1 (DOCX 6657 KB) [file 42995_2023_168_MOESM1_ESM.docx]

**Supporting material**

**Discovery of a natural small-molecule AMP-activated kinase activator that alleviates nonalcoholic steatohepatitis**

**This file includes:**

**CHNQD-0803** activates AMPK at a cellular level.

**CHNQD-0803** inhibits hepatocyte lipid accumulation.

**CHNQD-0803** alleviates cellular inflammation and improves hepatocyte fibrosis.

**CHNQD-0803** ameliorated hepatic steatosis, prevented hepatic inflammation, fibrosis and liver injury.

**CHNQD-0803** increased AMPK activity in NASH mice.

^1^H and ^13^C NMR spectrum of **CHNQD-0803**, **CHNQD-0811**, **CHNQD-0801**, **CHNQD-0802** and **CHNQD-0803a–m** in DMSO-*d*_6_.

**
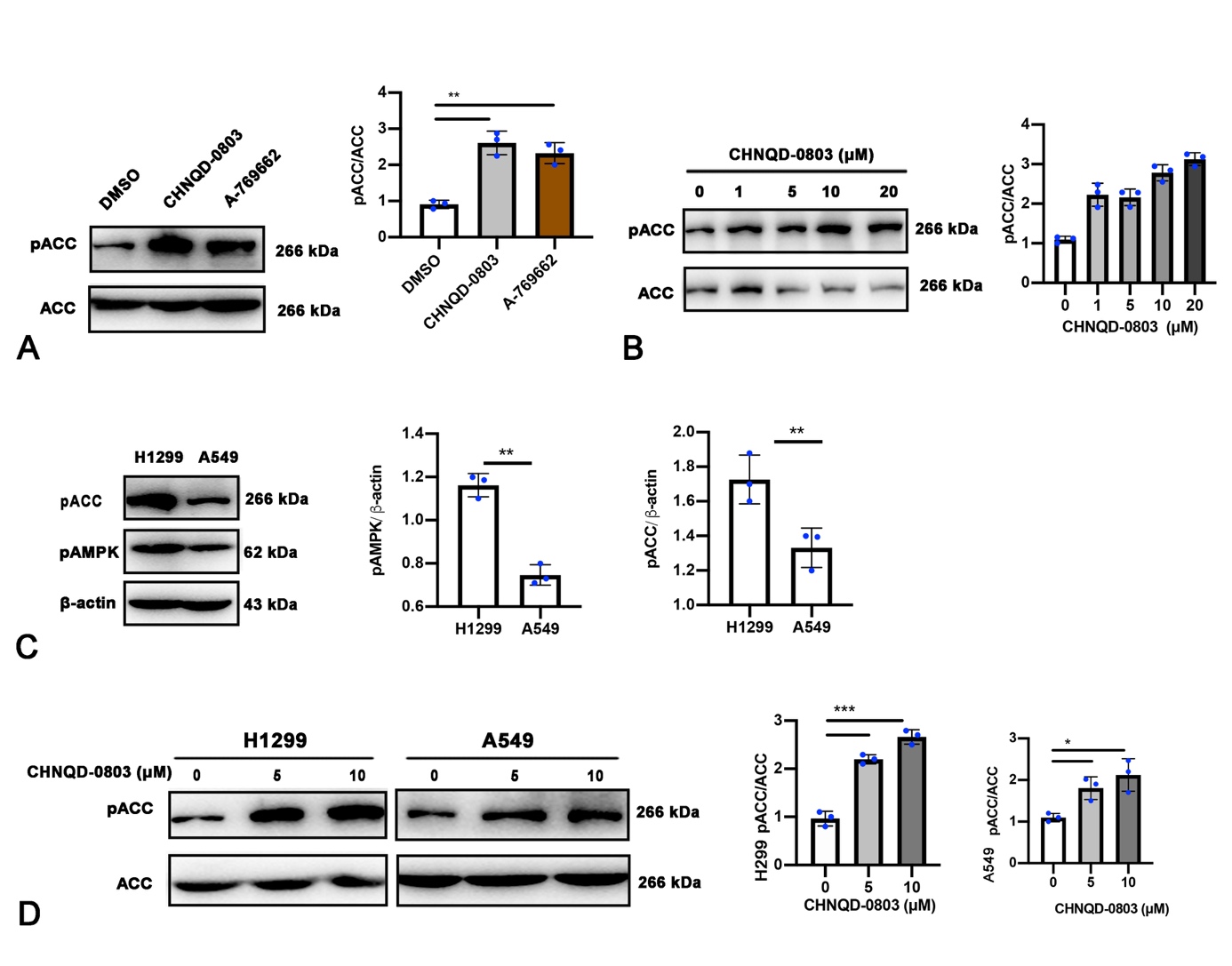
**

**Supplementary Fig. S1 CHNQD-0803 activates AMPK at a cellular level.**

(A) AMPK kinase activity of **CHNQD-0803** and other AMPK activator in HepG2 cells. HepG2 cells were treated with **CHNQD-0803** (10 μM) and A-769662 (10 μM) for 2 h and with DMSO as a control. (B) In HepG2 cells, a dose-dependent activation of AMPK and phosphorylation of ACC were observed. (C) The phosphorylation level of ACC and AMPK expression in H1299 and A549 cells. (D) AMPK kinase activity treated with **CHNQD-0803** at different concentrations for 2 h in H1299 and A549 cells. Results from at least three independent experiments. Data are expressed as the mean ± SD (*n* = 3 biologic replicates). *P* values: ns (not significant), **P*<0.05, ***P*<0.01, ****P*<0.001.

**
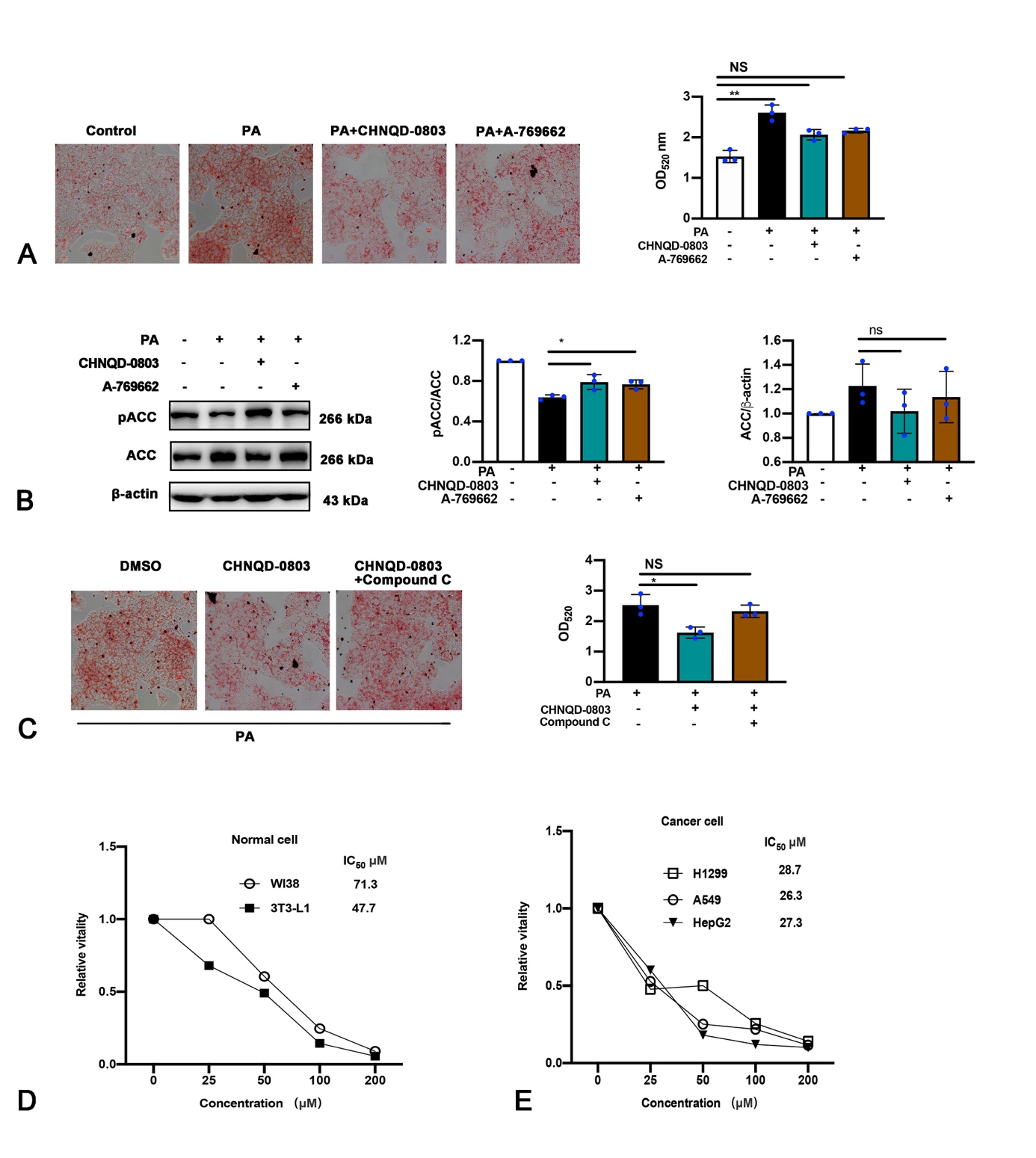
**

**Supplementary Fig. S2 CHNQD-0803 inhibits hepatocyte lipid accumulation.**

(A) Effect of **CHNQD-0803** on lipid accumulation in HepG2 cells. HepG2 cells were treated with PA (200 μmol/L) for 24 h, then treated with **CHNQD-0803** (10 μmol/L) or A-769662 (10 μmol/L) for 24 h, and with DMSO as a control. The level of lipid accumulation was detected by OD_520_ nm. (B) Western blot analysis of pACC, ACC and β-actin expression in HepG2 cells treated with **CHNQD-0803** and A-769662. (C) HepG2 cells were treated with PA (200 μmol/L) for 24 h, then treated with **CHNQD-0803** (10 μmol/L) or Compound C (5 μmol/L) for 24 h, and with DMSO as a control. (D) The toxicity of **CHNQD-0803** in cancer cells. H1299, A549 and HepG2 were treated with **CHNQD-0803** for 72 h respectively, MTT assays were carried out. (E) The toxicity of **CHNQD-0803** in normal cells. WI38 and 3T3-L1 were treated with compound 27 for 72 h respectively. Data are expressed as mean ± SD. *P* values: ns (not significant), **P*<0.05, ***P*<0.01, ****P*<0.001.

**
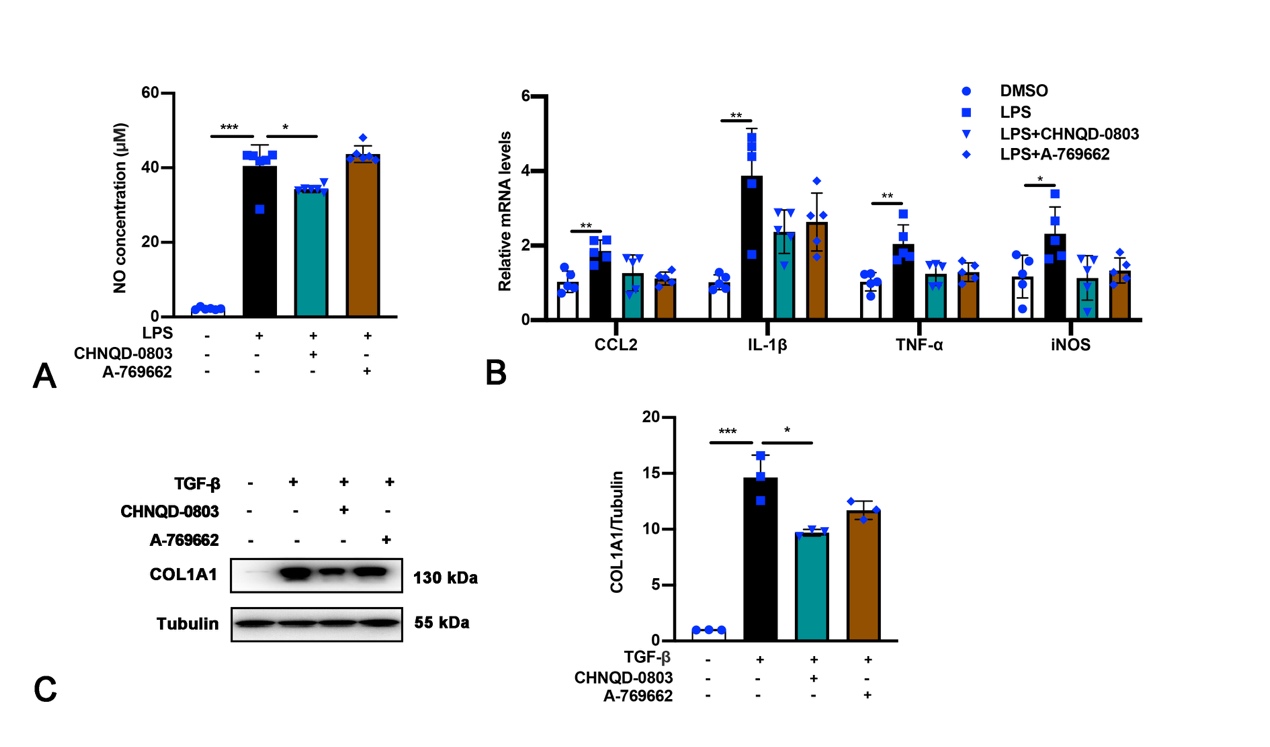
**

**Supplementary Fig. S3 CHNQD-0803 alleviates cellular inflammation and improves hepatocyte fibrosis.**

(A) RAW264.7 cells were treated with **CHNQD-0803** (10 μmol/L) or A-769662 (10 μmol/L) for 2 h, followed by challenge with LPS (100 ng/mL) for 24 h. The amount of NO in the supernatant was detected. (B) Total RNA was isolated, and the mRNA levels of TNF-α, IL-1β, CCL2 and iNOS were then quantified in RAW264.7 cells. (C) LX-2 cells were treated with TGF-β (10 ng/mL) for 12 h, then treated with **CHNQD-0803** (10 μmol/L) or A-769662 (10 μmol/L) for 24 h. Western blot analysis of COL1A1 and Tubulin expression in LX-2 cells. Data are expressed as mean ± SD. *P* values: ns (not significant), **P*<0.05, ***P*<0.01, ****P*<0.001.

**
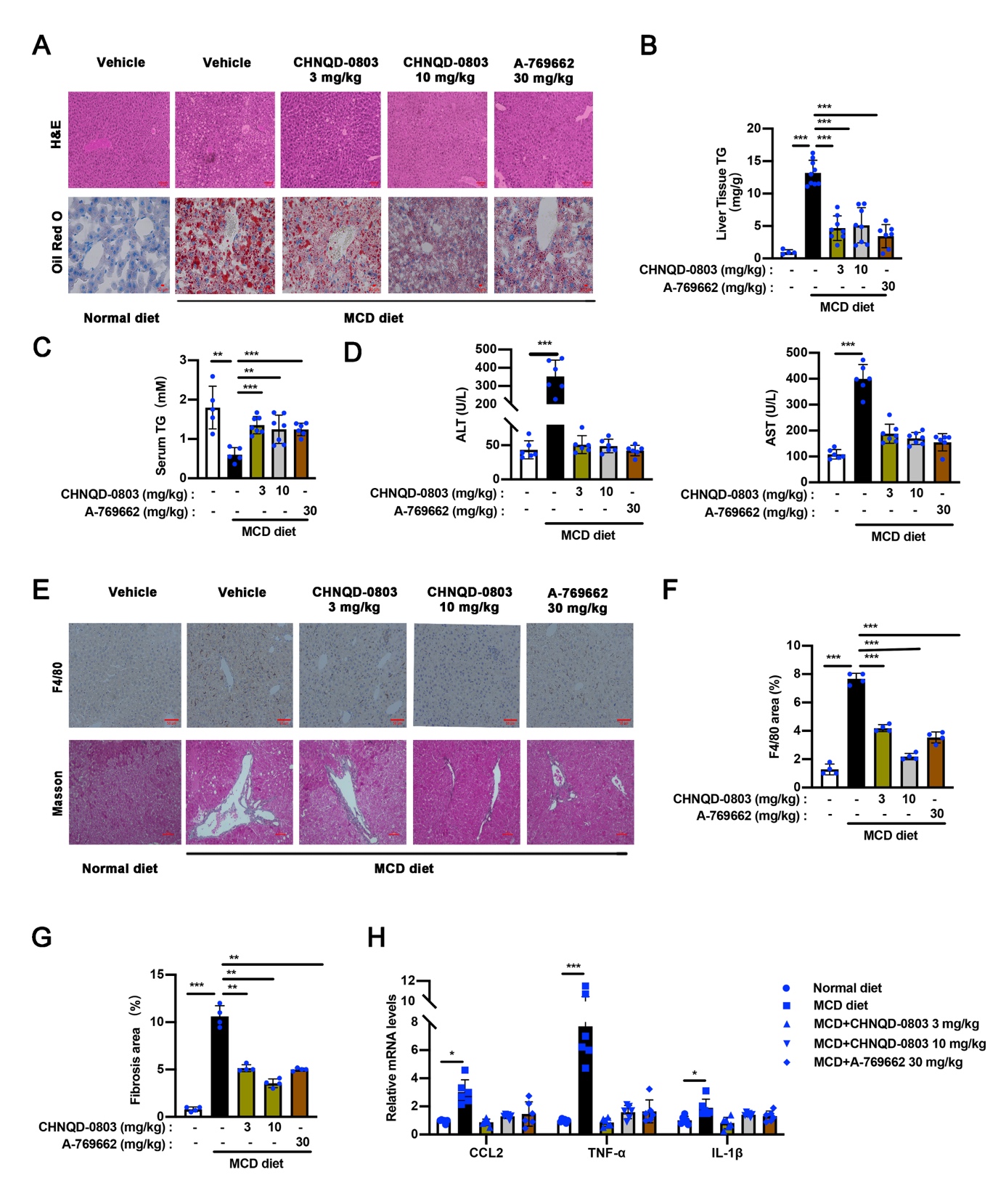
**

**Supplementary Fig. S4 CHNQD-0803 ameliorated hepatic steatosis, prevented hepatic inflammation, fibrosis and liver injury.**

(A) Liver images of mice stained with Hematoxylin-eosin and Oil Red O. (B) The level of hepatic triglyceride (TG) were tested after treatment of NASH mice with **CHNQD-0803** and A-769662. (C) The levels of TG in serum were detected. (D) The levels of blood biochemistry in serum, including ALT and AST were quantified using semiautomatic biochemistry analyzer. (E) Immunohistochemical staining results of F4/80 (yellow dots) on mice liver sections and representative images of mice liver sections stained with Masson (blue). (F) The expression level of F4/80 in mice liver were analyzed. (G) The area of collagen fibers in livers of mice were analyzed. (H) mRNA levels of the indicated genes in NASH mice liver. Data are expressed as mean ± SD. (*n* = 6 mice per group). *P* values: ns (not significant), **P*<0.05, ***P*<0.01, ****P*<0.001.


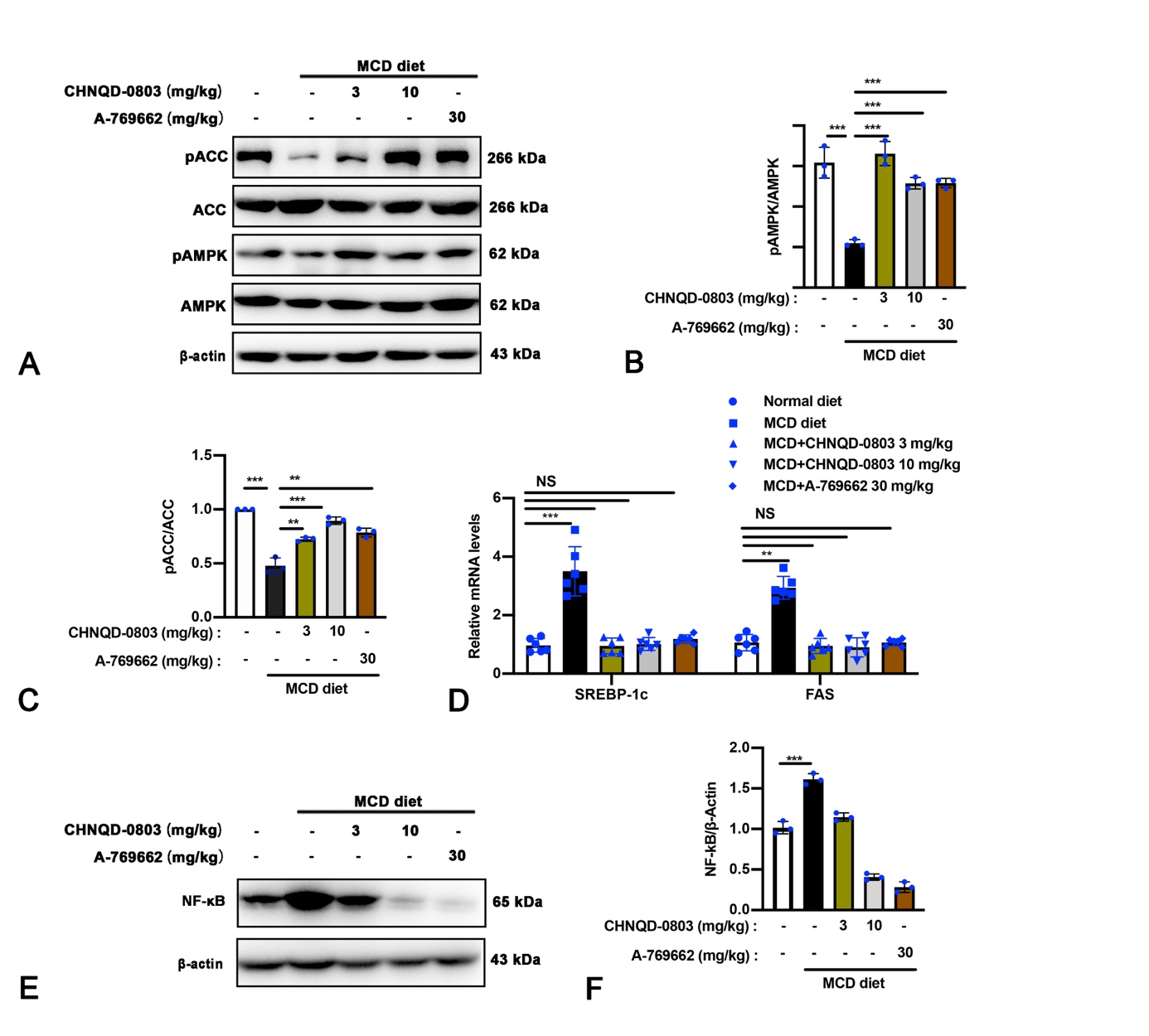


**Supplementary Fig. S5 CHNQD-0803 increased AMPK activity in NASH mice.**

(A) Western blot detection of ACC, pACC, AMPK and pAMPK expression in the liver after treatment of NASH mice with **CHNQD-0803** and A-769662. (B, C) pAMPK/AMPK and pACC/ACC were quantified. (D) mRNA levels of the indicated genes in NASH mice liver were analyzed. (E) Western blot detection of NF-κB expression in the liver after treatment of NASH mice with **CHNQD-0803** and A-769662. (F) NF-κB/β-actin was quantified. Data are expressed as mean ± SD. (*n* = 6 mice per group). *P* values: ns (not significant), **P*<0.05, ***P*<0.01, ****P*<0.001.


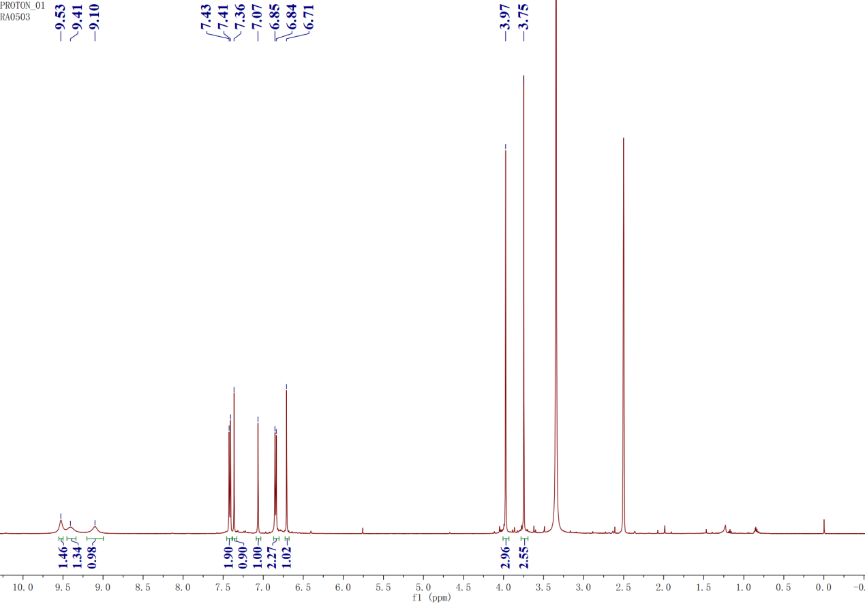


**Supplementary Fig. S6**  ^1^H NMR spectrum of **CHNQD-0803** in DMSO-*d*_6_.


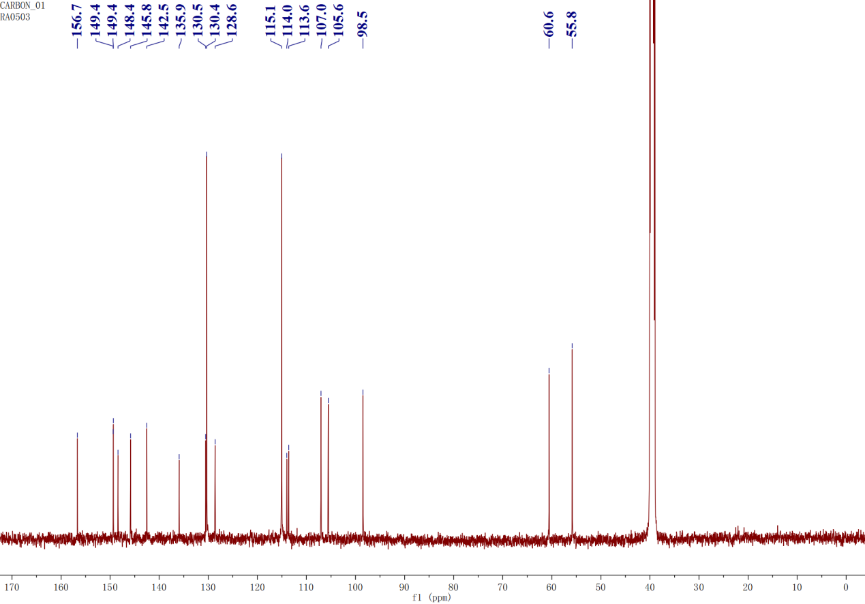


**Supplementary Fig. S7** ^13^C NMR spectrum of **CHNQD-0803** in DMSO-*d*_6.、_


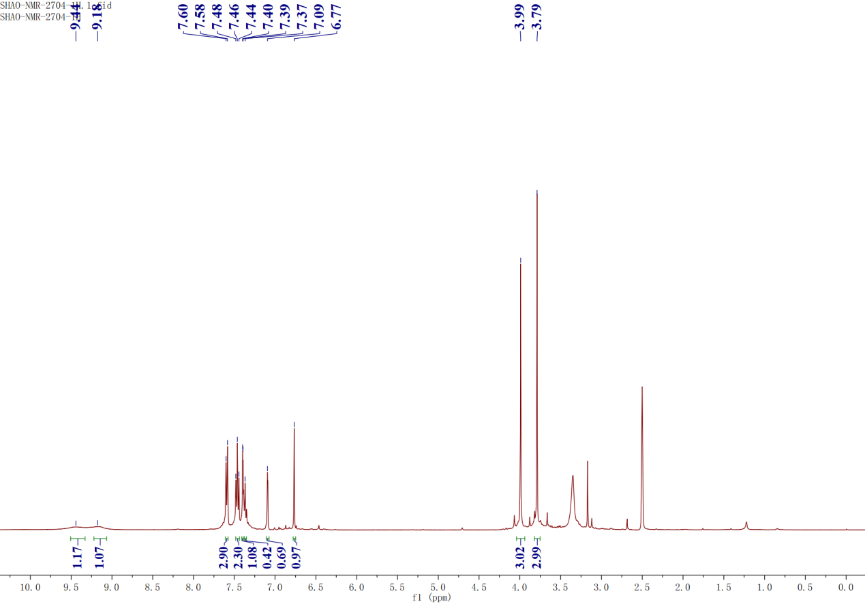


**Supplementary Fig. S8** ^1^H NMR spectrum of **CHNQD-0811** in DMSO-*d*_6_.


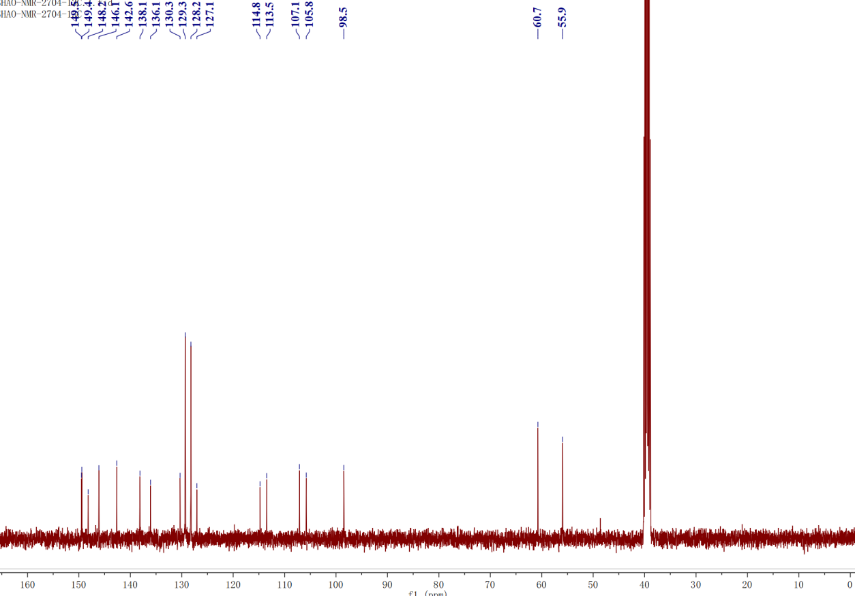
 **Supplementary Fig. S9** ^13^C NMR spectrum of **CHNQD-0811** in DMSO-*d*_6._

_
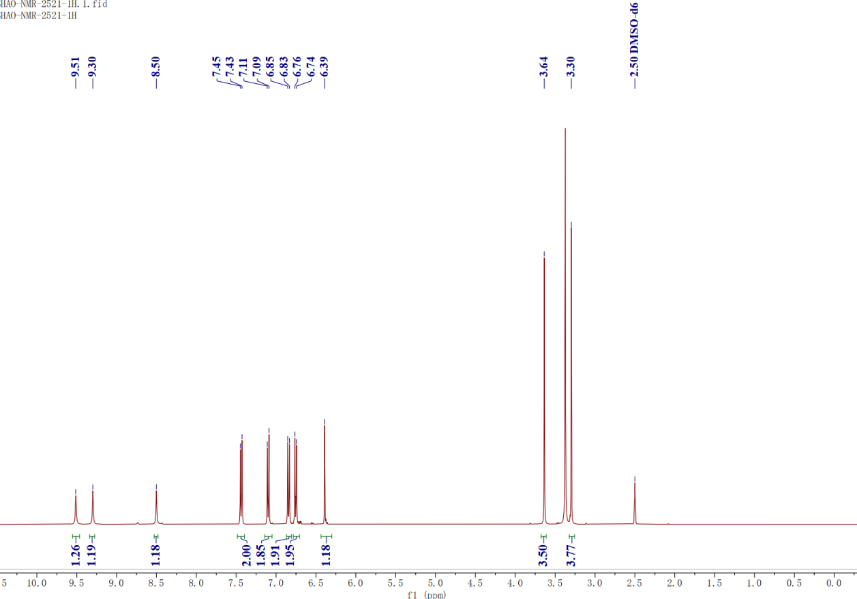
_

**Supplementary Fig. S10** ^1^H NMR spectrum of **CHNQD-0801** in DMSO-*d*_6_.


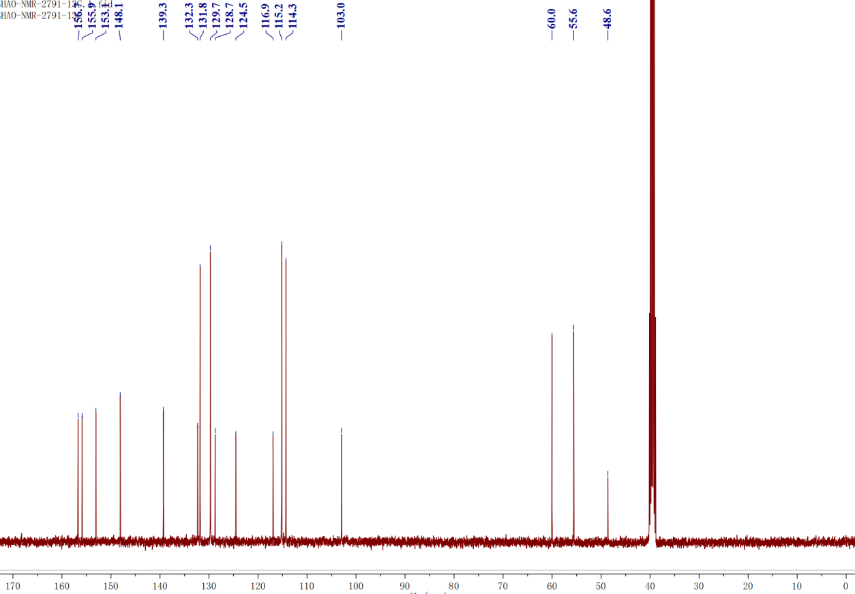
 **Supplementary Fig. S11** ^13^C NMR spectrum of **CHNQD-0801** in DMSO-*d*_6._


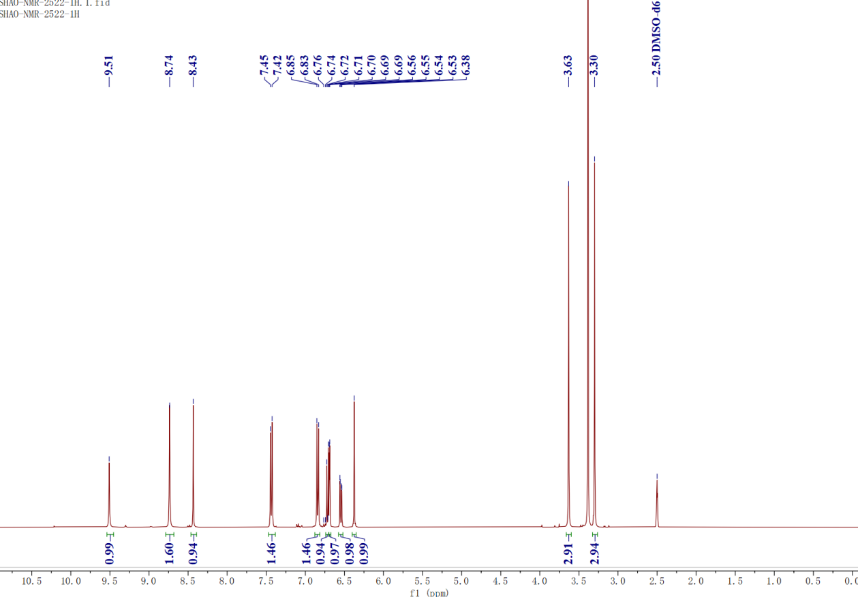


**Supplementary Fig. S12** ^1^H NMR spectrum of **CHNQD-0802** in DMSO-*d*_6_.


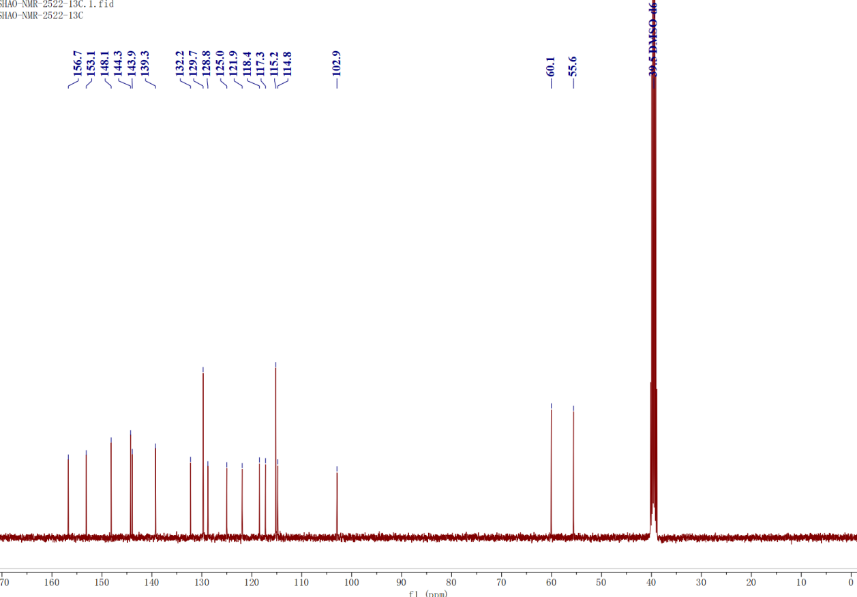


**Supplementary Fig. S13**^13^C NMR spectrum of **CHNQD-0802** in DMSO-*d*_6._

**Supplementary Fig. S14** HRESIMS spectrum of **CHNQD-0803a**.


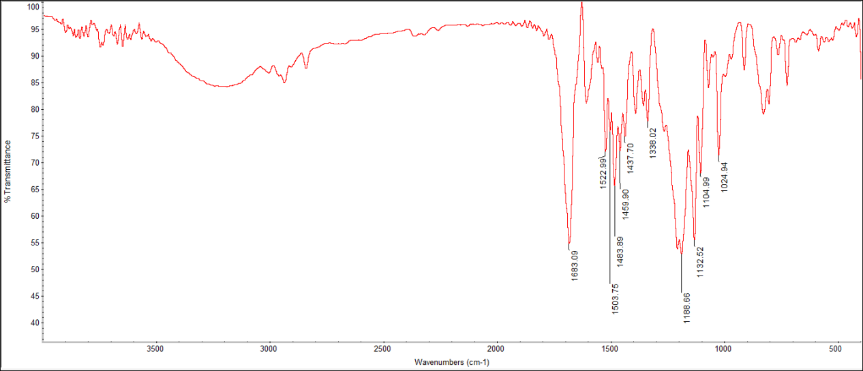


**Supplementary Fig. S15** IR spectrum of **CHNQD-0803a**.


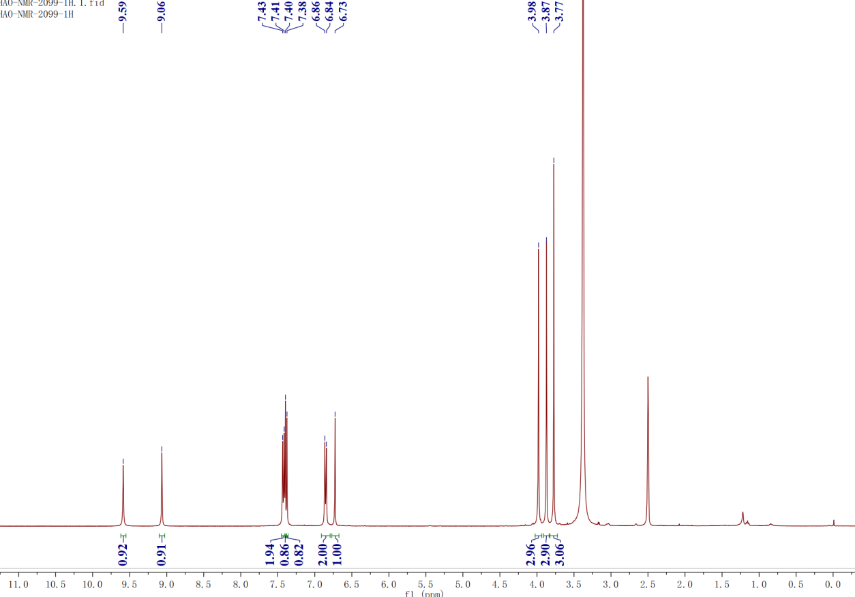


**Supplementary Fig. S16.** ^1^H NMR spectrum of **CHNQD-0803a** in DMSO-*d*_6_.


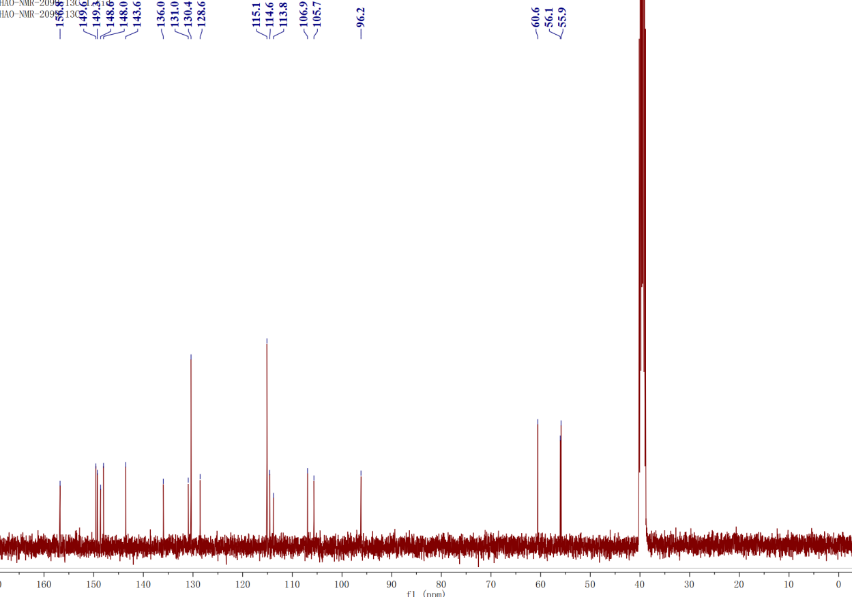


**Supplementary FigS17.**^13^C NMR spectrum of **CHNQD-0803a** in DMSO-*d*_6._

 **Supplementary Fig. S18.** HRESIMS spectrum of **CHNQD-0803b**.


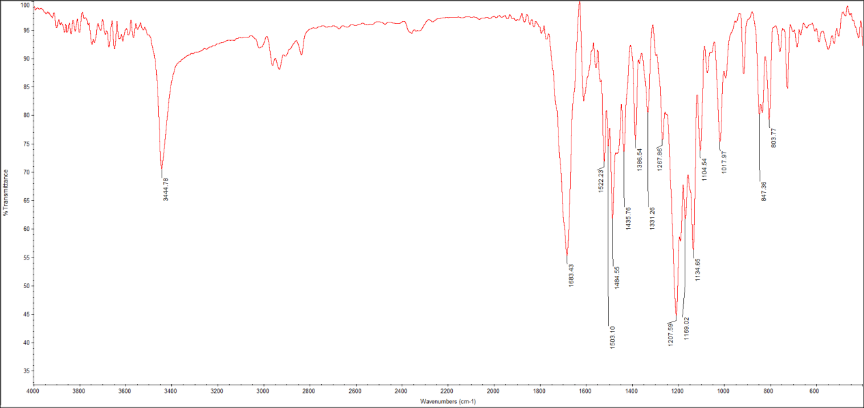


**Supplementary Fig. S19.** IR spectrum of **CHNQD-0803b**.


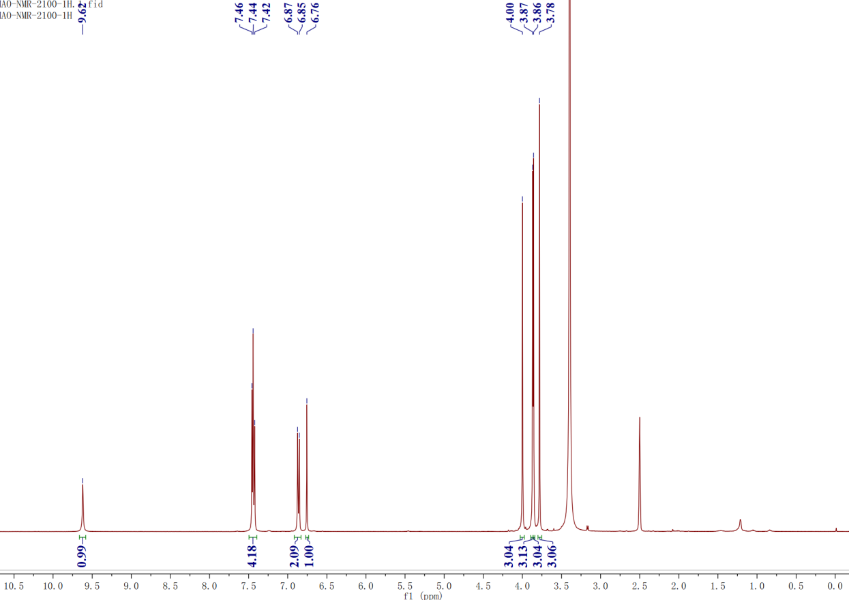


**Supplementary Fig. S20.** ^1^H NMR spectrum of **CHNQD-0803b** in DMSO-*d*_6_.

**
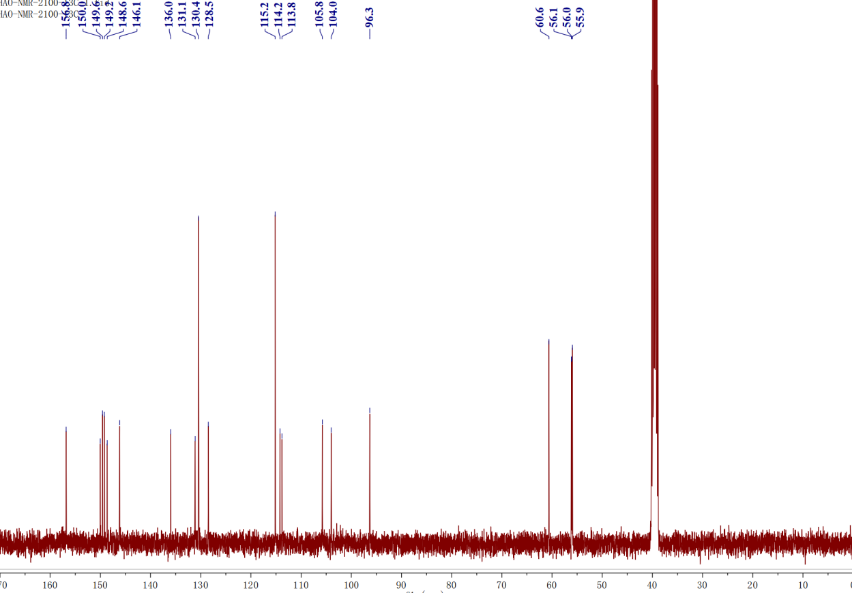
**

**Supplementary Fig. S21.**^13^C NMR spectrum of **CHNQD-0803b** in DMSO-*d*_6._

 **Supplementary Fig. S22.** HRESIMS spectrum of **CHNQD-0803c**.


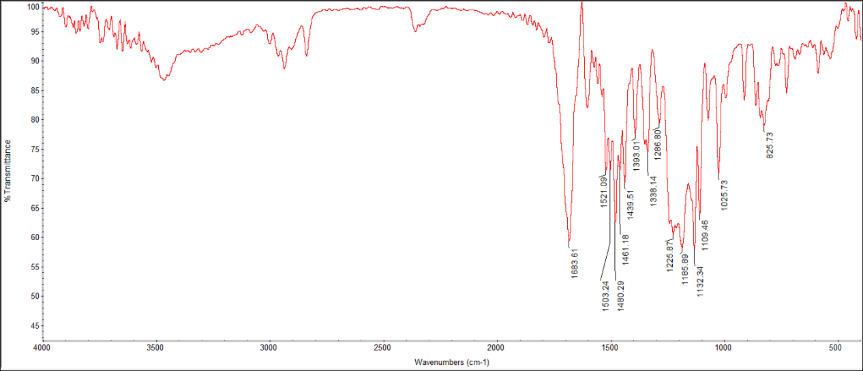


**Supplementary Fig. S23.** IR spectrum of **CHNQD-0803c**.

**
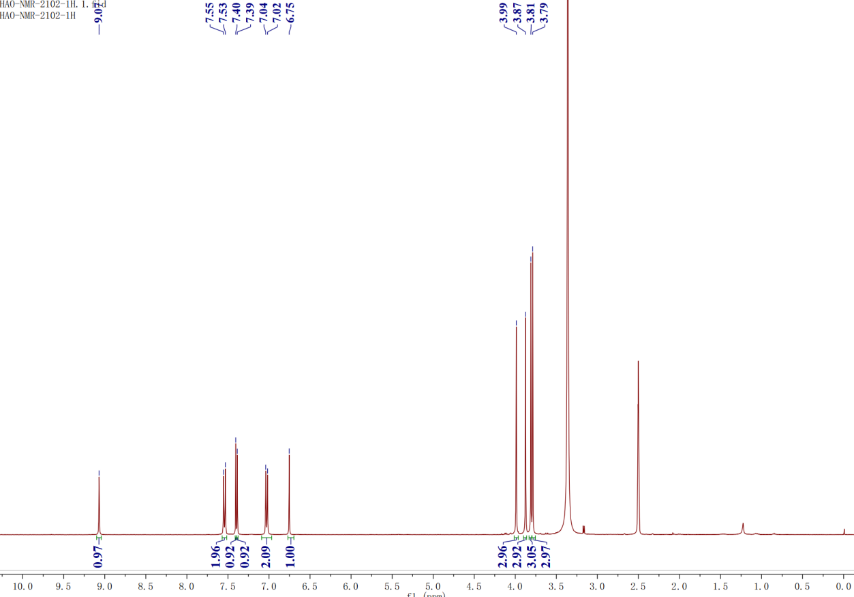
**

**Supplementary Fig. S24.** ^1^H NMR spectrum of **CHNQD-0803c** in DMSO-*d*_6_.


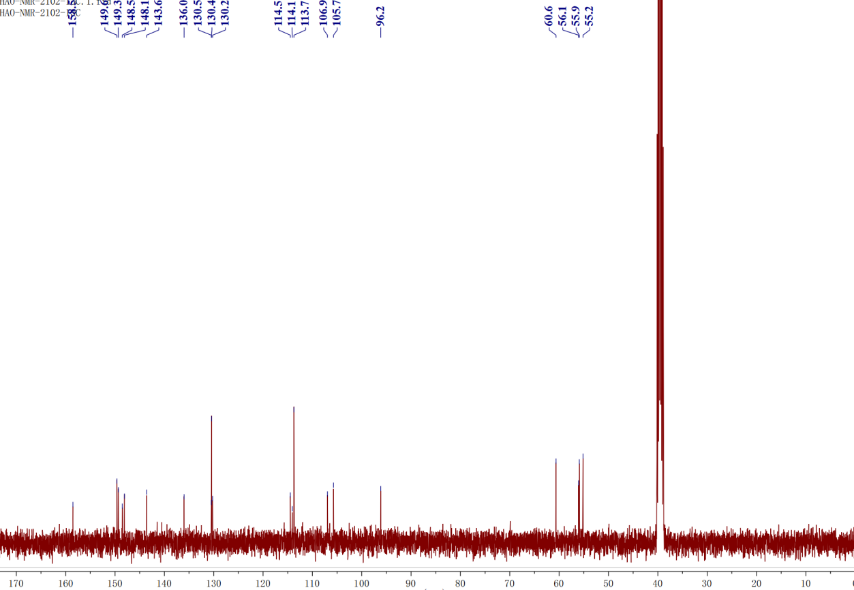


**Supplementary Fig. S25.**^13^C NMR spectrum of **CHNQD-0803c** in DMSO-*d*_6._

 **Supplementary Fig. S26.** HRESIMS spectrum of **CHNQD-0803d**.


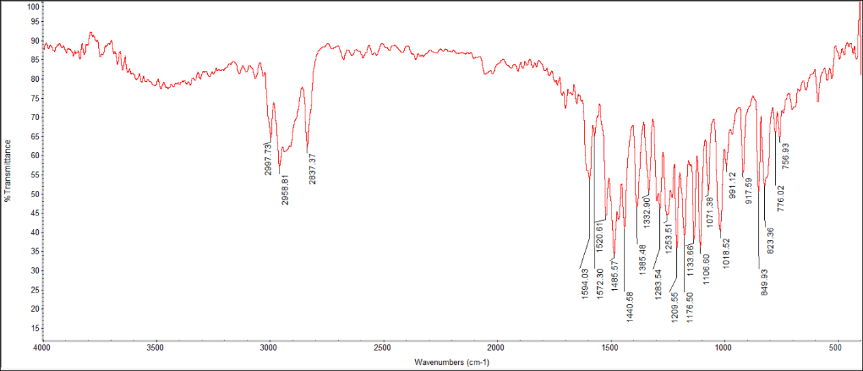


**Supplementary Fig. S27.** IR spectrum of **CHNQD-0803d**.

**
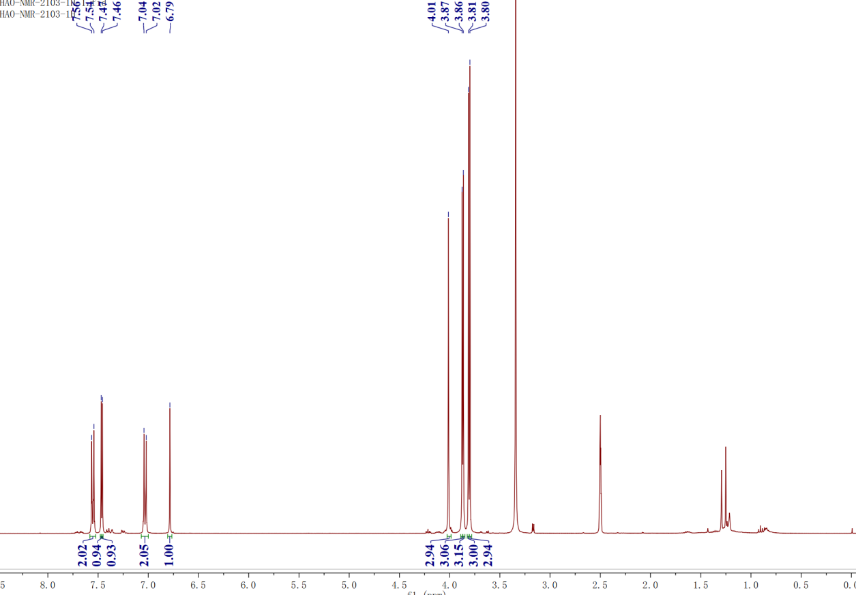
**

**Supplementary Fig. S28.** ^1^H NMR spectrum of **CHNQD-0803d** in DMSO-*d*_6_.

**
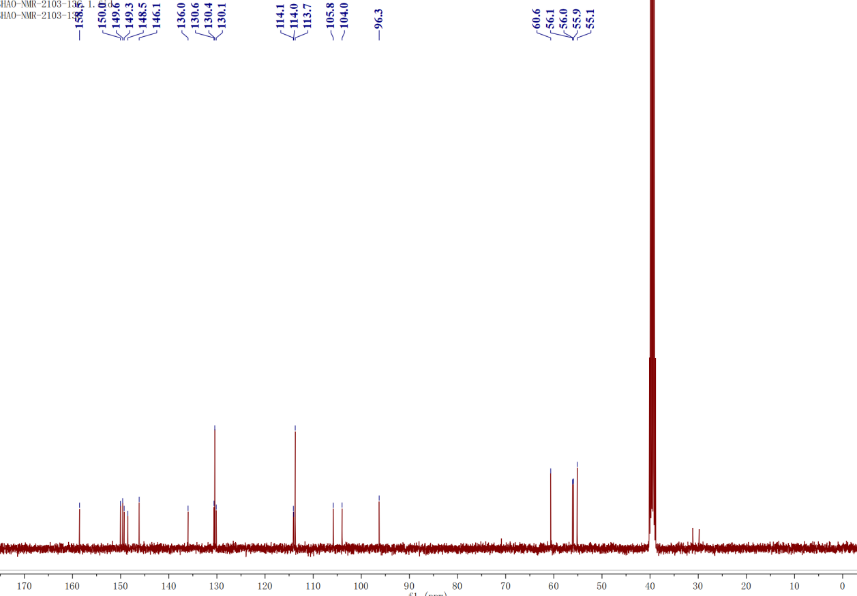
**

**Supplementary Fig. S29.**^13^C NMR spectrum of **CHNQD-0803d** in DMSO-*d*_6._

**Supplementary Fig. S30.** HRESIMS spectrum of **CHNQD-0803e**.


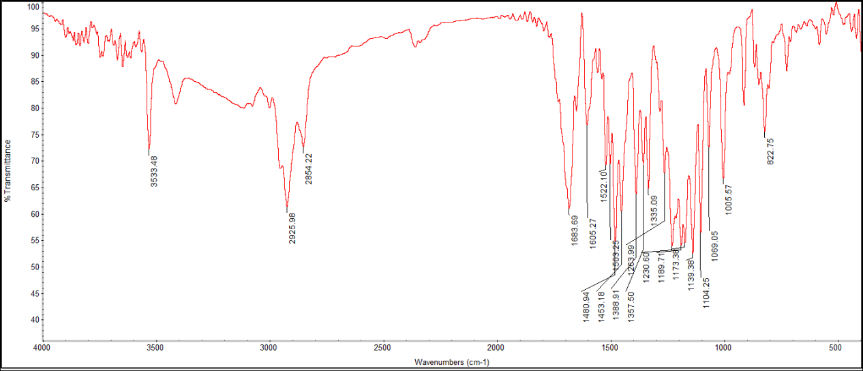


**Supplementary Fig. S31.** IR spectrum of **CHNQD-0803e**.


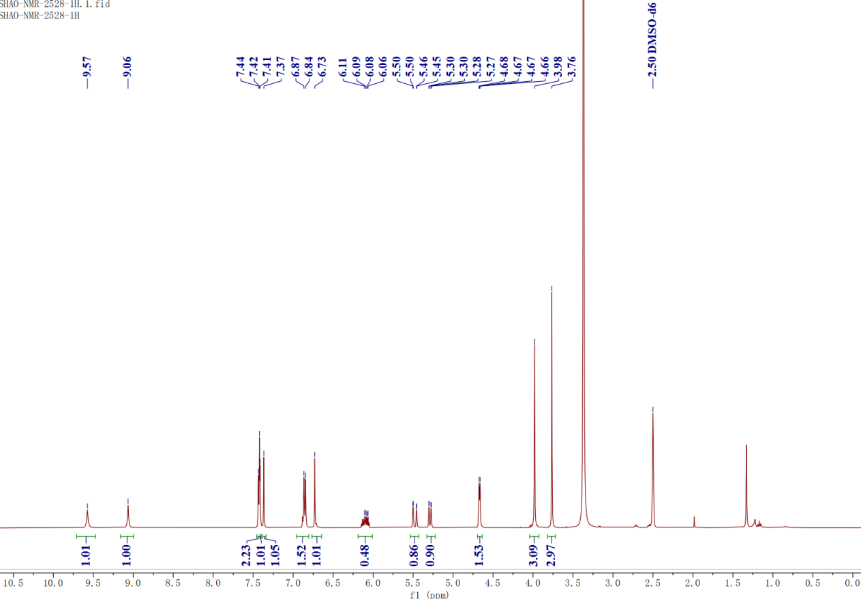


**Supplementary Fig. S32.** ^1^H NMR spectrum of **CHNQD-0803e** in DMSO-*d*_6_.


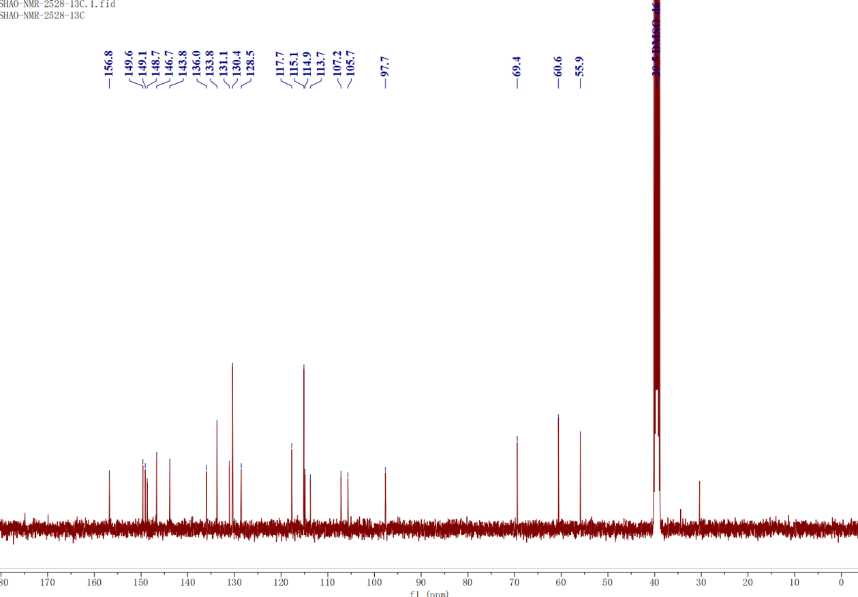


**Supplementary Fig. S33.**^13^C NMR spectrum of **CHNQD-0803e** in DMSO-*d*_6._

**Supplementary Fig. S34.** HRESIMS spectrum of **CHNQD-0803f**.


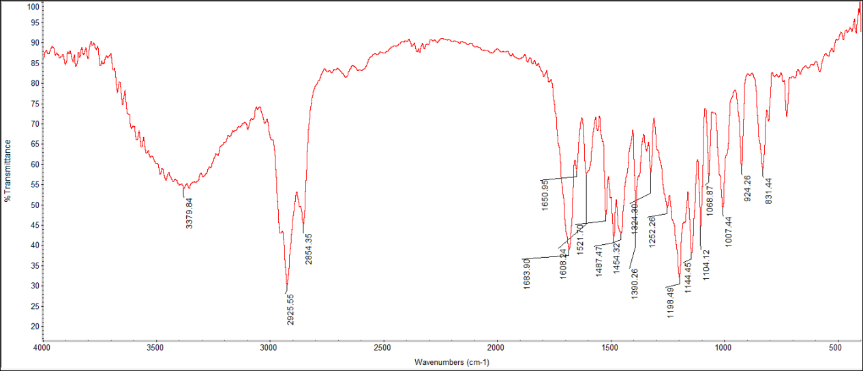


**Supplementary Fig. S35.** IR spectrum of **CHNQD-0803f**.


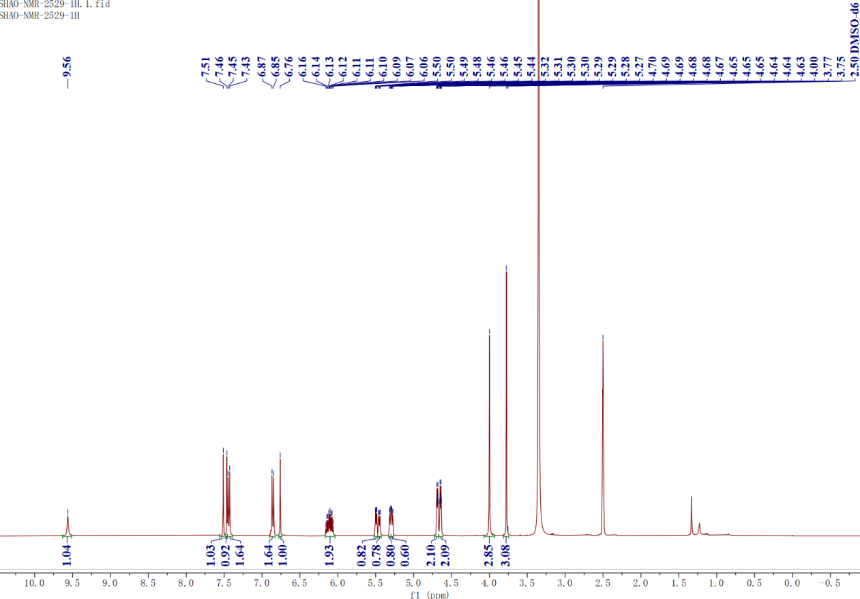


**Supplementary Fig. S36.** ^1^H NMR spectrum of **CHNQD-0803f** in DMSO-*d*_6_.


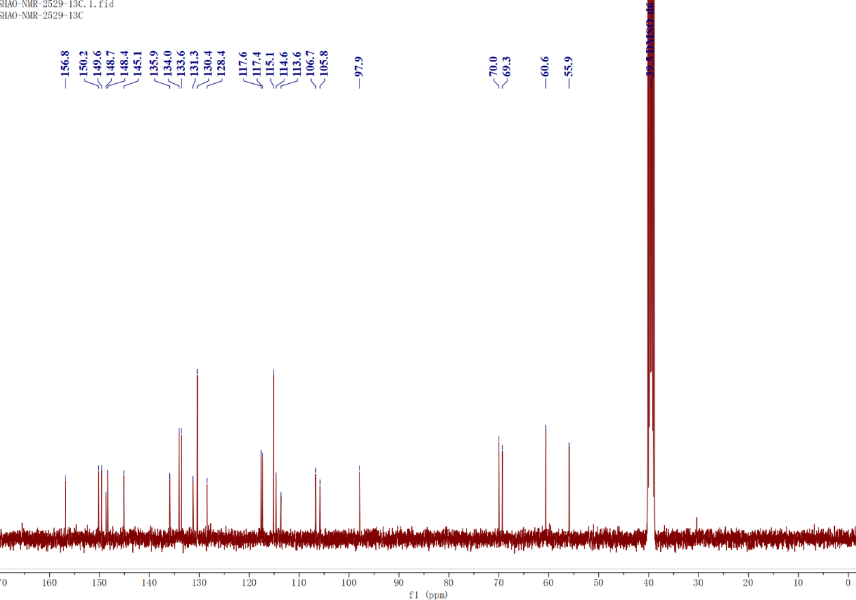


**Supplementary Fig. S37.**^13^C NMR spectrum of **CHNQD-0803f** in DMSO-*d*_6._

 **Supplementary Fig. S38.** HRESIMS spectrum of **CHNQD-0803g**.


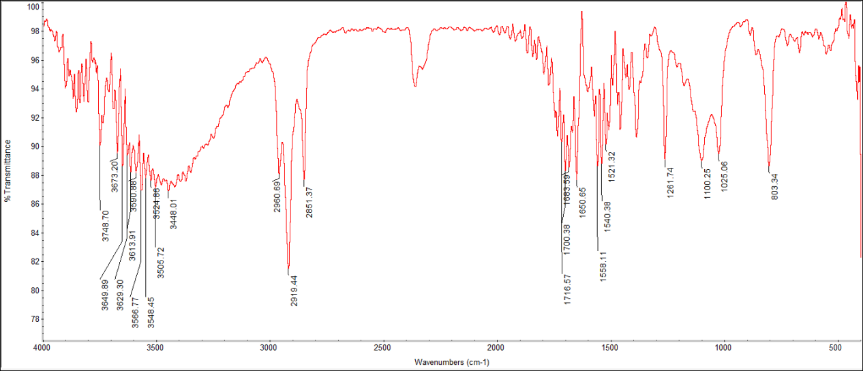


**Supplementary Fig. S39.** IR spectrum of **CHNQD-0803g**.


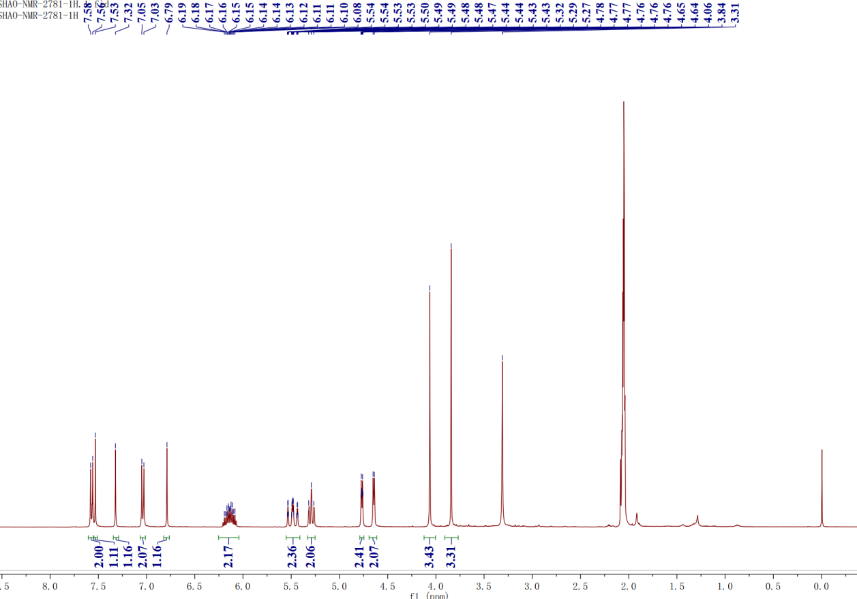


**Supplementary Fig. S40.** ^1^H NMR spectrum of **CHNQD-0803g** in acetone-*d*_6_.

**
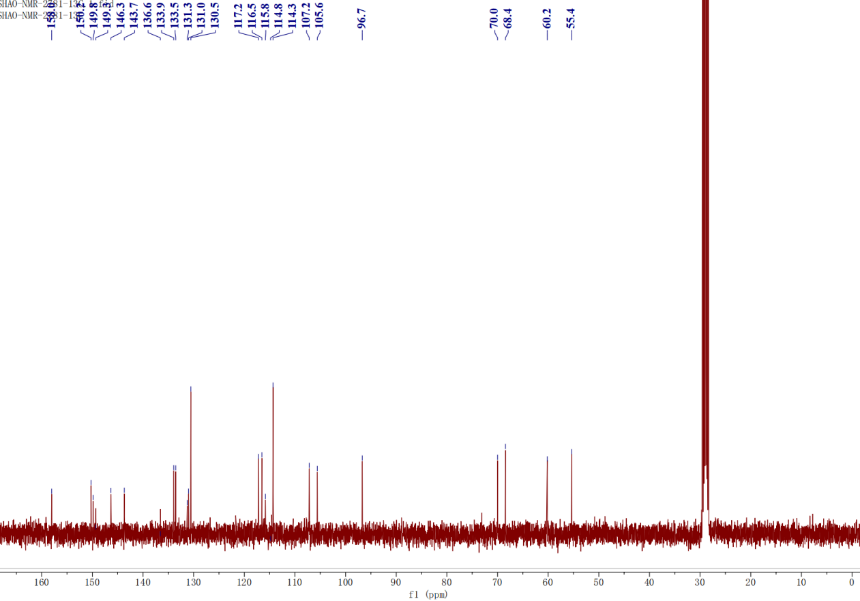
 Supplementary Fig. S41.** ^13^C NMR spectrum of **CHNQD-0803g** in acetone-*d*_6_.

**Supplementary Fig. S42.** HRESIMS spectrum of **CHNQD-0803h**.


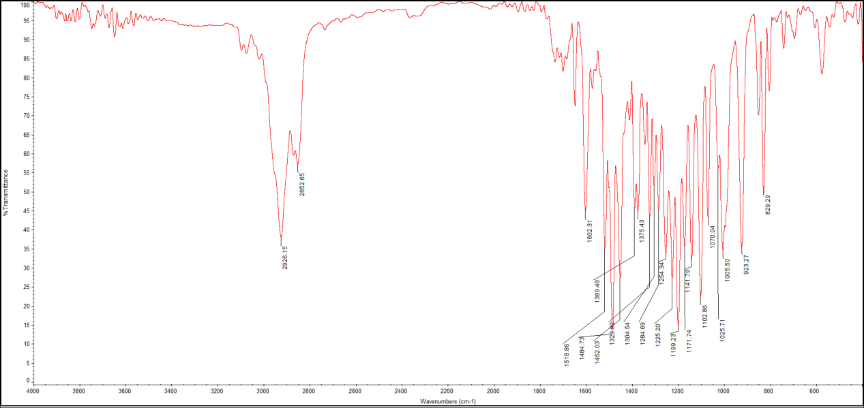


**Supplementary Fig. S43.** IR spectrum of **CHNQD-0803h**.


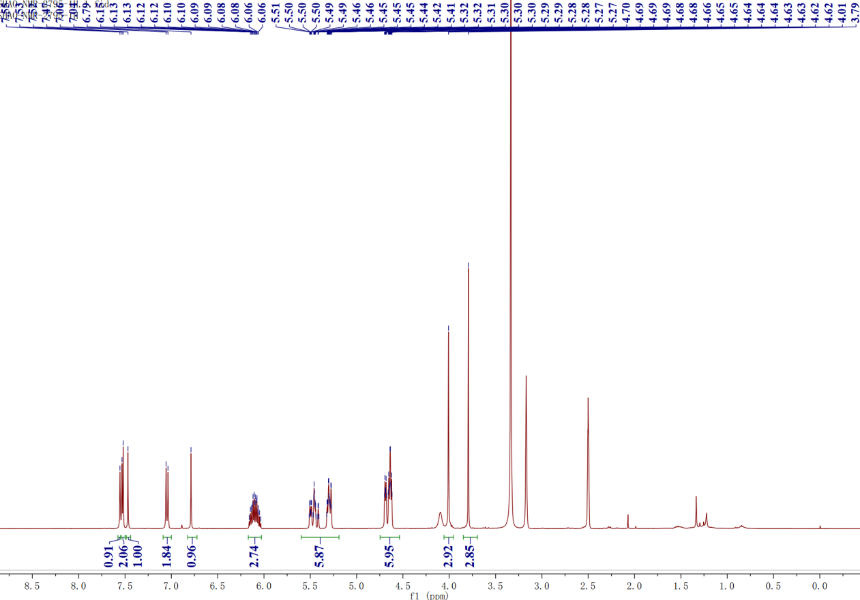


**Supplementary Fig. S44.** ^1^H NMR spectrum of **CHNQD-0803h** in DMSO-*d*_6_.


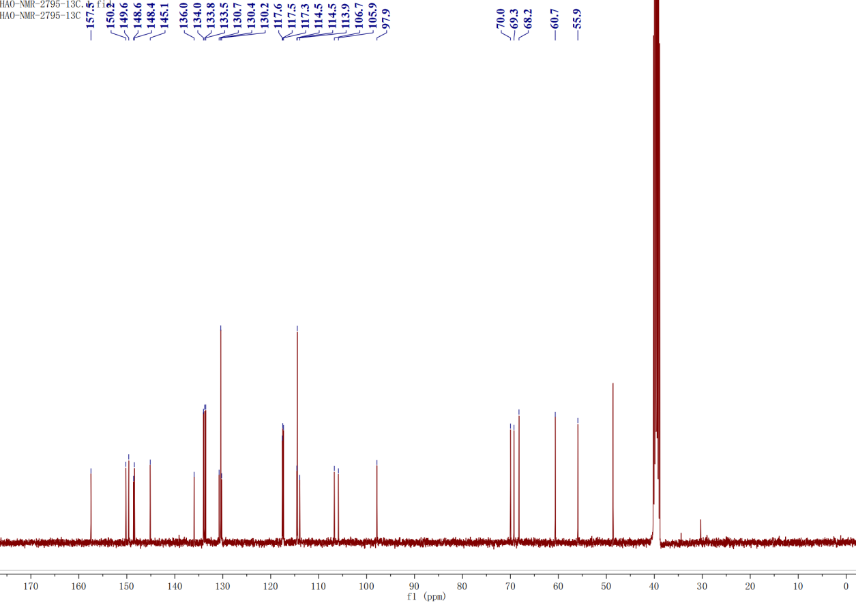


**Supplementary Fig. S45.** ^1^H NMR spectrum of **CHNQD-0803h** in DMSO-*d*_6_.

**Supplementary Fig. S46.** HRESIMS spectrum of **CHNQD-0803i**.


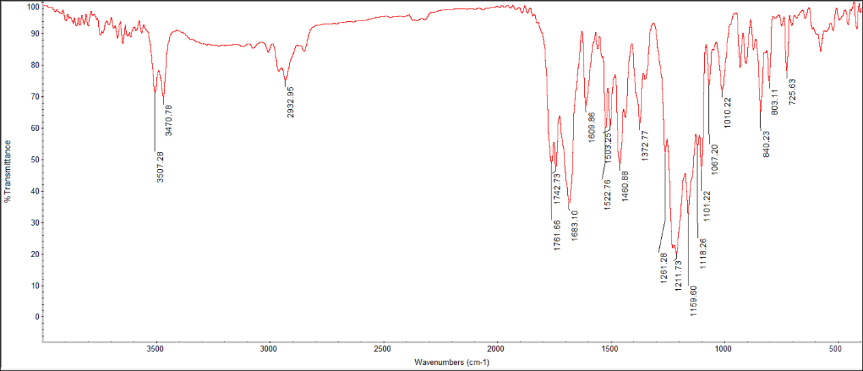


**Supplementary Fig. S47.** IR spectrum of **CHNQD-0803i**.


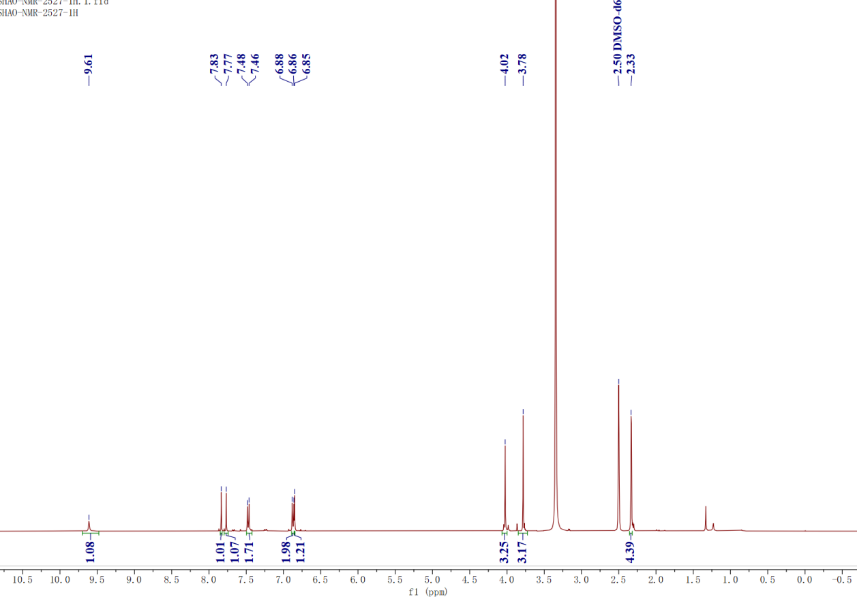


**Supplementary Fig. S48.** ^1^H NMR spectrum of **CHNQD-0803i** in DMSO-*d*_6_.

**
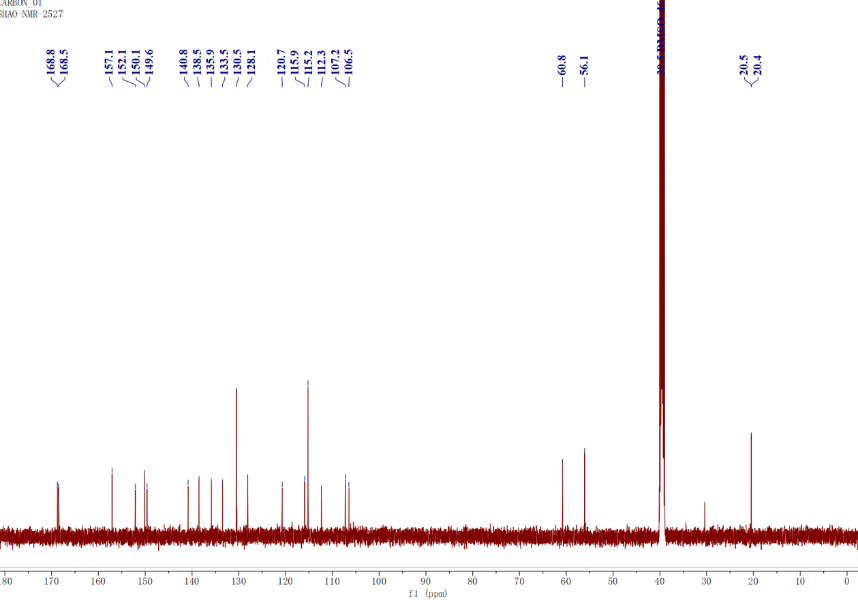
**

**Supplementary Fig. S49.**^13^C NMR spectrum of **CHNQD-0803i** in DMSO-*d*_6._

 **Supplementary Fig. S50.** HRESIMS spectrum of **CHNQD-0803j**.


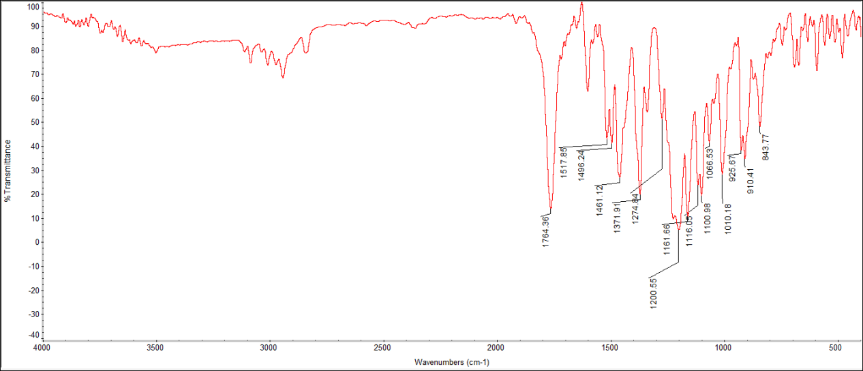


**Supplementary Fig. S51.** IR spectrum of **CHNQD-0803j**.

**
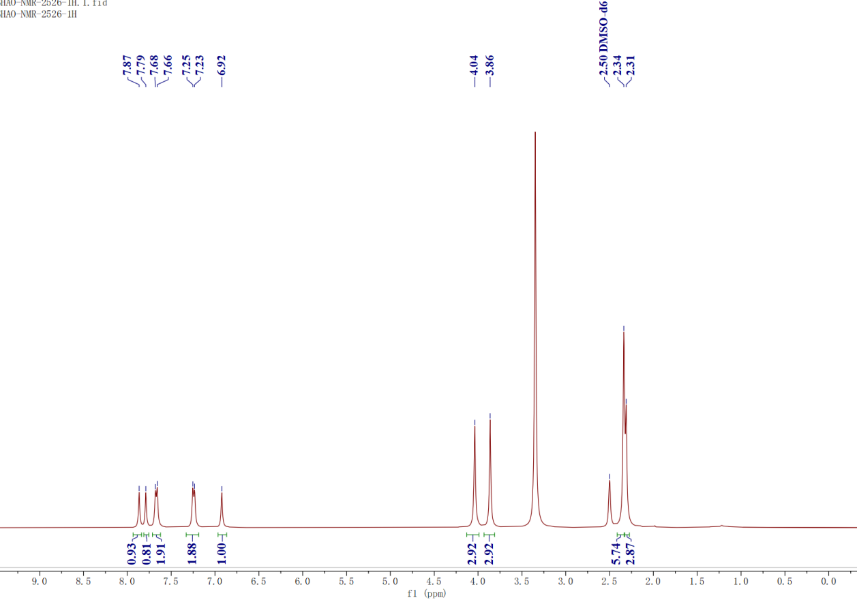
**

**Supplementary Fig. S52.** ^1^H NMR spectrum of **CHNQD-0803j** in DMSO-*d*_6_.

**
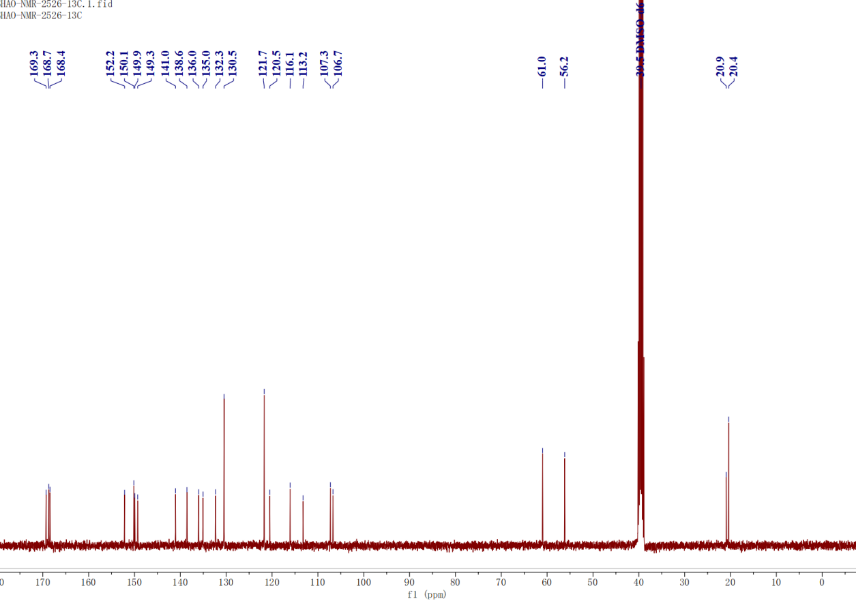
**

**Supplementary Fig. S53.**^13^C NMR spectrum of **CHNQD-0803j** in DMSO-*d*_6._

 **Supplementary Fig. S54.** HRESIMS spectrum of **CHNQD-0803k**.


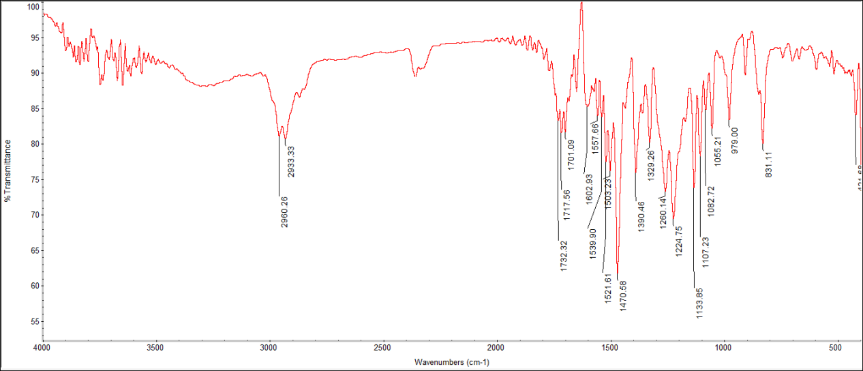


**Supplementary Fig. S55.** IR spectrum of **CHNQD-0803k**.

**
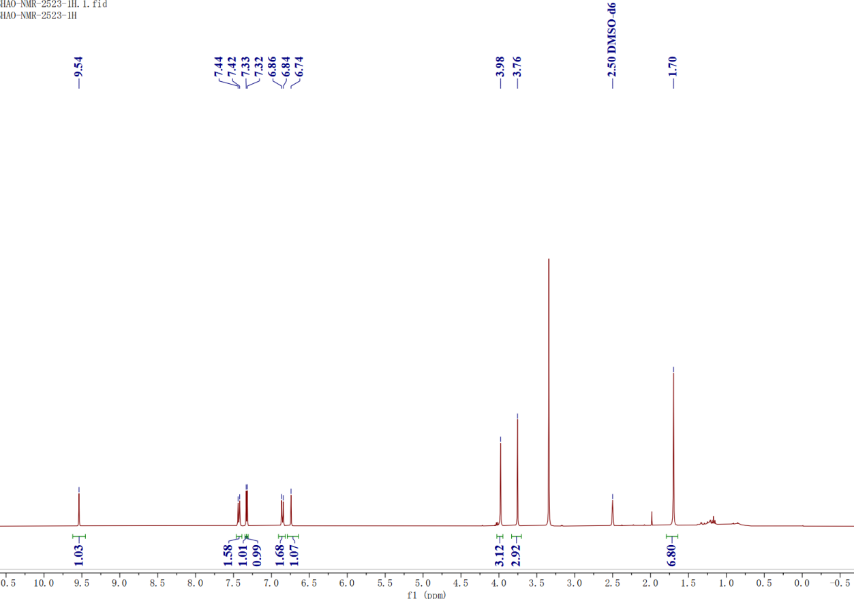
**

**Supplementary Fig. S56.** ^1^H NMR spectrum of **CHNQD-0803k** in DMSO-*d*_6_.

**
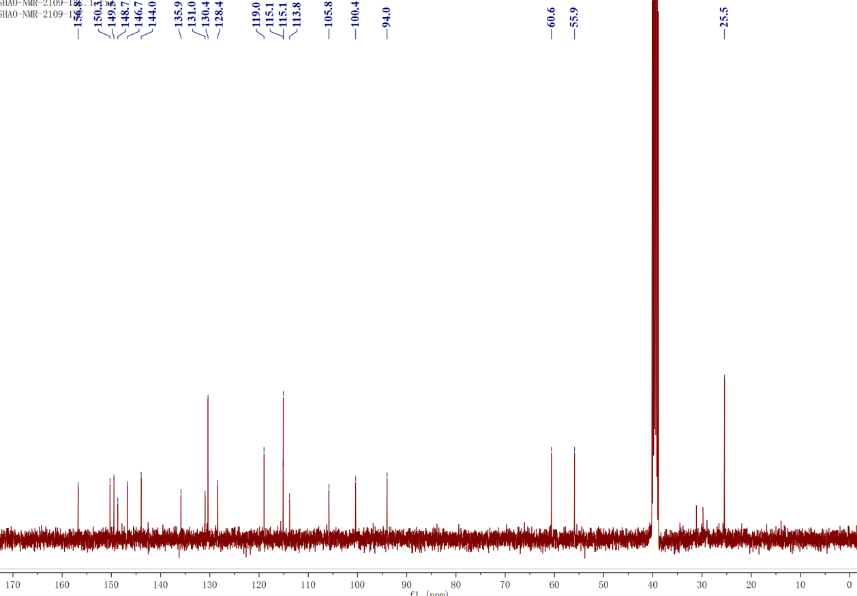
**

**Supplementary Fig. S57.**^13^C NMR spectrum of **CHNQD-0803k** in DMSO-*d*_6._

 **Supplementary Fig. S58.** HRESIMS spectrum of **CHNQD-0803l**.


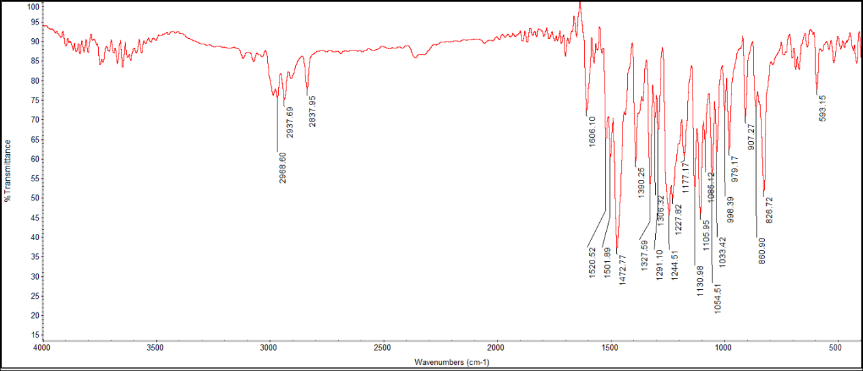


**Supplementary Fig. S59.** IR spectrum of **CHNQD-0803l**.


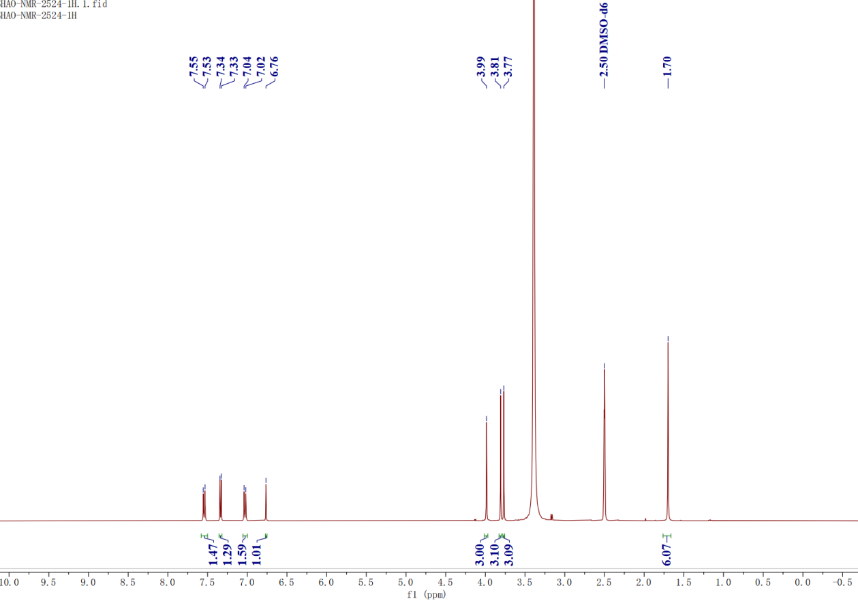


**Supplementary Fig. S60.** ^1^H NMR spectrum of **CHNQD-0803l** in DMSO-*d*_6_.


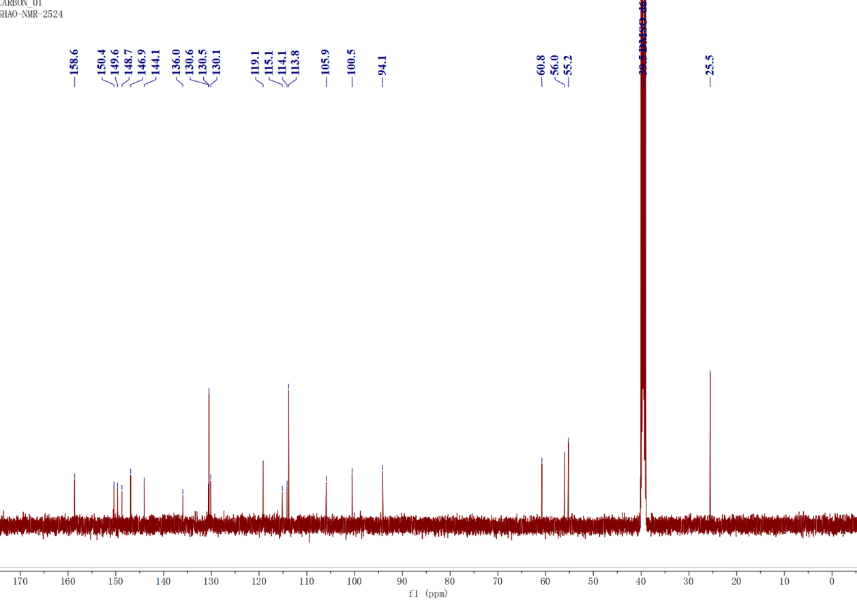


**Supplementary Fig. S61.**^13^C NMR spectrum of **CHNQD-0803l** in DMSO-*d*_6._

 **Supplementary Fig. S62.** HRESIMS spectrum of **CHNQD-0803m**.

_
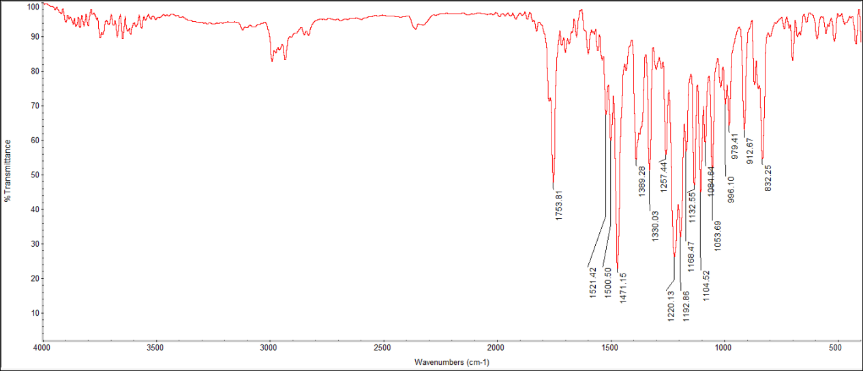
_ **Supplementary Fig. S63.** IR spectrum of **CHNQD-0803m**.


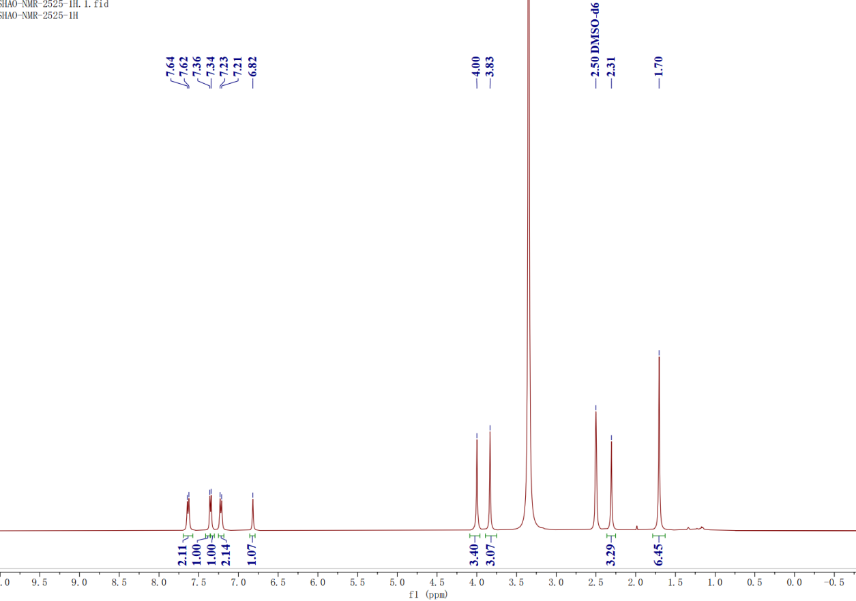
 **Supplementary Fig. S64.** ^1^H NMR spectrum of **CHNQD-0803m** in DMSO-*d*_6_.


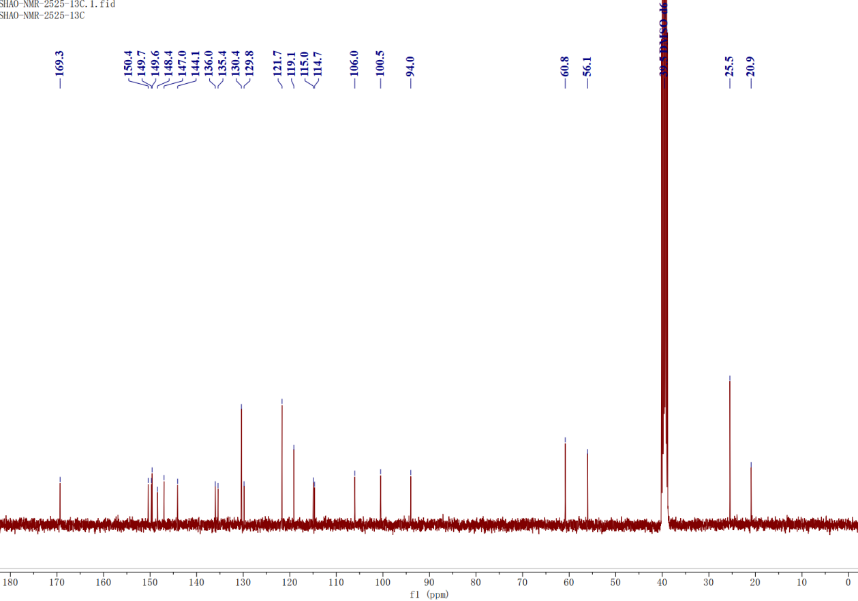


**Supplementary Fig. S65.**^13^C NMR spectrum of **CHNQD-0803m** in DMSO-*d*_6._
